# A quantitative systems pharmacology (QSP) platform for preclinical to clinical translation of *in-vivo* CRISPR-Cas therapy

**DOI:** 10.3389/fphar.2024.1454785

**Published:** 2024-09-20

**Authors:** Devam A. Desai, Stephan Schmidt, Rodrigo Cristofoletti

**Affiliations:** Center of Pharmacometrics and Systems Pharmacology, University of Florida, Orlando, FL, United States

**Keywords:** quantitative systems pharmacology, gene therapy, liver disease, rare disease, translational modeling

## Abstract

**Background:** In-vivo CRISPR Cas genome editing is a complex therapy involving lipid nanoparticle (LNP), messenger RNA (mRNA), and single guide RNA (sgRNA). This novel modality requires prior modeling to predict dose-exposure-response relationships due to limited information on sgRNA and mRNA biodistribution. This work presents a QSP model to characterize, predict, and translate the Pharmacokinetics/Pharmacodynamics (PK/PD) of CRISPR therapies from preclinical species (mouse, non-human primate (NHP)) to humans using two case studies: transthyretin amyloidosis and LDL-cholesterol reduction.

**Methods:** PK/PD data were sourced from literature. The QSP model incorporates mechanisms post-IV injection: 1) LNP binding to opsonins in liver vasculature; 2) Phagocytosis into the Mononuclear Phagocytotic System (MPS); 3) LNP internalization via endocytosis and LDL receptor-mediated endocytosis in the liver; 4) Cellular internalization and transgene product release; 5) mRNA and sgRNA disposition via exocytosis and clathrin-mediated endocytosis; 6) Renal elimination of LNP and sgRNA; 7) Exonuclease degradation of sgRNA and mRNA; 8) mRNA translation into Cas9 and RNP complex formation for gene editing. Monte-Carlo simulations were performed for 1000 subjects and showed a reduction in serum TTR.

**Results:** The rate of internalization in interstitial layer was 0.039 1/h in NHP and 0.007 1/h in humans. The rate of exocytosis was 6.84 1/h in mouse, 2690 1/h in NHP, and 775 1/h in humans. Pharmacodynamics were modeled using an indirect response model, estimating first-order degradation rate (0.493 1/d) and TTR reduction parameters in NHP.

**Discussion:** The QSP model effectively characterized biodistribution and dose-exposure relationships, aiding the development of these novel therapies. The utility of platform QSP model can be paramount in facilitating the discovery and development of these novel agents.

## Introduction

In the past decade, the field of genomic medicine has undergone a transformative change with the advent of clustered regularly interspaced short palindromic repeats (CRISPR) and CRISPR-associated protein (Cas) systems (Jinek et al., 2012; Abdelhady et al., 2023). These systems, originally identified as an adaptive immune mechanism in bacteria, have paved the way for precise, programmable genome editing, revolutionizing the potential for curative therapies in human disease (Jinek et al., 2012). The transition of CRISPR-Cas from a basic research tool to a clinical therapeutic modality has marked a significant milestone in the pursuit of treating genetic disorders at their root cause.

The development of *in vivo* CRISPR therapies represents a cutting-edge frontier in medicine, offering a direct and efficient method to correct genetic defects directly within the body. Unlike respective *ex vivo* approaches, which require cell extraction, modification, and reinfusion, *in vivo* CRISPR therapies involve the systemic delivery of the gene-editing components, targeting the cells in their natural physiological environment. This approach not only broadens the therapeutic potential to a wider range of diseases, including those affecting organs and tissues difficult to access *ex vivo*, but also promises a more streamlined and potentially less invasive treatment option (Abdelhady et al., 2023; Çerçi et al., 2023).

Both preclinical studies and clinical trials have shown promising results in utilizing CRISPR-Cas systems for *in vivo* applications (Çerçi et al., 2023). The ability to directly correct or modify genes within the patient’s body circumvents the complexities and limitations associated with cell extraction and reinfusion processes, opening new avenues for treating a plethora of genetic conditions.

Furthermore, the advancements in delivery mechanisms, such as lipid nanoparticles (LNPs), have significantly enhanced the precision in terms of targeting the liver or any other target organ of interest by an antibody-based LNP system, efficiency, and safety of these *in vivo* gene-editing therapies, making them a viable option for clinical applications (Kumar et al., 2023).

*In vivo* CRISPR therapies are administered *via* intravenous infusion. They consist of three components: 1) delivery vehicle, 2) single guide RNA (sgRNA), and 3) messenger RNA (mRNA). For the delivery vehicle, both the LNP and adeno-associated virus (AAV) are used. Changes in the delivery vehicle will impact the pharmacokinetics (PK) of CRISPR therapies (Abdelhady et al., 2023; Kavita et al., 2023; Suzuki and Ishihara, 2021; Akinc et al., 2010). In this paper, we focus on the LNP as the delivery vehicle encapsulating the sgRNA and mRNA product.

Gene editing *via* sgRNA and mRNA is accomplished by the following sequential steps: 1) post-injection entering into the liver; 2) internalization of the bound LNP via receptor-mediated endocytosis binding to a low-density lipoprotein (LDL) receptor via apolipoprotein E (ApoE) present on the surface of the LNP or unbound LNP via macropinocytosis (Miyazawa et al., 2024; Paunovska et al., 2022; Bisgaier et al., 1989); 3) entering into the cellular layer and releasing the sgRNA and mRNA product via endosomal escape (Hou et al., 2021); 4) mRNA getting translated into Cas protein of interest (Abdelhady et al., 2023; Miyazawa et al., 2024); 5) sgRNA and Cas protein will form a ribonucleoprotein (RNP) complex to perform gene editing by entering the nucleus, where it recognizes the protospacer adjacent motif (PAM) on the complementary DNA strand, the sgRNA binds to the target site, and Cas9 undergoes conformational changes, leading to DNA cleavage (Abdelhady et al., 2023; Li et al., 2019; Rouet and Christ, 2019); 6) the sgRNA and mRNA might also escape from the cell via exocytosis and can get internalized back via clathrin-dependent endocytosis or caveola-mediated endocytosis and macropinocytosis (Wu and Li, 2021; Del Toro Runzer et al., 2023; Desai et al., 2019); and 7) the sgRNA, mRNA, and LNP exhibit lysosomal degradation (Fujiwara et al., 2016).

To our knowledge, there is no prior model published that characterizes and predicts the *in vivo* PK/pharmacodynamics (PD) of CRISPR-Cas9 therapies across species due to the novelty of these therapeutics, their unique biodistribution, and their complex mechanism-of-action. As a result, there is a need to develop a translational platform quantitative systems pharmacology (QSP) model that can capture these processes across multiple species to inform the development of *in vivo* CRISPR therapies from preclinical species to the clinic and to predict the first-in-human (FIH) dosing (Shah and Betts, 2012; Betts et al., 2019; Ayyar and Song, 2024; Ayyar et al., 2021; Singh et al., 2021; Mager and Jusko, 2001; Mager and Jusko, 2008). This objective is approached in the current paper in a stepwise fashion, starting with the extraction of available PK-PD studies in mice, non-human primates (NHPs), and humans from the literature. We developed a translational QSP model to characterize the whole body to intracellular PK-PD actions of CRISPR-Cas therapy and physiologically scaled the platform model across species. In order to evaluate the impact of drug-specific parameters on the biodistribution of *in vivo* CRISPR-Cas therapy across species, a global sensitivity analysis (GSA) was conducted. Monte Carlo simulations were performed to understand and characterize the dose–response relationship for NTLA-2001 *in vivo* CRISPR-Cas therapy in patients suffering from transthyretin amyloidosis (Kotit, 2023).

## Methods

### Model development

The data used to build the translational QSP model for *in vivo* CRISPR-Cas9 gene therapy were obtained from the published literature, as given in Table 1. The PK data for NTLA-2001 in mice were obtained from a published study (Finn et al., 2018). The reported dataset for mice includes mean pharmacokinetic measurements in plasma for mRNA and sgRNA, sampled at 0, 0.2, 0.4, 0.6, 0.8, 1, 10, and 25 h and quantified using the quantitative polymerase chain reaction (qPCR) assay. The mice were dosed with a bolus dose of 2 mg/kg total RNA, in which 33.3% and 66.7% were calculated to be sgRNA and mRNA, and the total LNP dose was calculated to be 36.7 mg/kg based on the published source (Gillmore et al., 2021).

**TABLE 1:** Summary of published preclinical and clinical datasets which are used in order to develop the proposed translational QSP model.

Target	Total RNA dose (mg/kg)	Species	Measurement(s)	Reference(s)
TTR	2 (IV bolus)	Mouse	sgRNA and mRNA plasma PK	Finn et al. (2018)
TTR	1–3 (IV infusion)	NHP	LNP plasma PK	Gillmore et al. (2021)
TTR	1.5–6 (IV infusion)	NHP	Serum TTR	Gillmore et al. (2021)
LDL cholesterol	0.75–1.5 (IV infusion)	NHP	Serum PCSK9 and serum LDL cholesterol	Lee et al. (2023)
TTR	0.1–1 (IV infusion)	Human	LNP plasma PK	Abdelhady et al. (2023)
TTR	0.1–1 (IV infusion)	Human	Serum TTR	Gane et al. (2022)

TTR, transthyretin; PCSK9, proprotein convertase subtilisin/kexin type 9; LNP, lipid nanoparticle; mRNA, messenger mRNA; sgRNA, single guide RNA; LDL, low-density lipoprotein.

The PK for the lipid nanoparticle of NTLA-2001 targeting transthyretin (TTR) amyloidosis in NHPs has been reported following a 2-h infusion of 1, 2, or 3 mg/kg total RNA, in which 33.3% and 66.7% was sgRNA and mRNA, respectively, and the total LNP doses were 18.5, 36.7, and 55.5 mg/kg based on a published report (Gillmore et al., 2021). The plasma PK was sampled at 1.5, 4, and 8 h in cynomolgus monkeys. The biomarkers for this drug include the TTR protein; the concentration of TTR was quantified using enzyme-linked immunosorbent assay (ELISA); the biomarker response for NTLA-2001 was reported for a short-term infusion of 2 h for doses of 1.5, 3, and 6 mg/kg total RNA; and the total LNP doses were 27.75, 68.82, and 137.64 mg/kg (Gillmore et al., 2021). The biomarkers for a second case study for the investigational drug VERVE-101 include PCSK9 measured in blood and LDL cholesterol (Lee et al., 2023). The response was reported for a short-term infusion of 2 h for doses of 0.75 and 1.5 mg/kg of total RNA and 17.2 and 27.75 mg/kg of LNP calculated based on the published literature (Gillmore et al., 2021). The PCSK9 sampled in blood and LDL cholesterol was quantified using a commercial immunoassay kit (Protein Simple Ella Simple Plex Human PCSK9) and Beckman Coulter AU680 analyzer, respectively (Lee et al., 2023).

The mean plasma pharmacokinetics of the LNP for NTLA-2001 was reported in humans following a short-term infusion of 2 h for doses of 0.1, 0.3, 0.7, and 1 mg/kg of total RNA and 1.85, 5.55, 12.9, and 18.5 mg/kg of LNP calculated based on the published literature (Abdelhady et al., 2023), and the pharmacokinetic data for the LNP were sampled from 0 to 50 h (Abdelhady et al., 2023). The TTR biomarker response was digitized from the literature for NTLA-2001, and TTR reduction was sampled at 7, 14, and 28 days (Gane et al., 2022).

### Model structure and workflow

A stepwise approach was applied to develop and validate the platform model using data leveraged from *in vivo* studies in mice and NHPs, and clinical responses in humans (Shah and Betts, 2012; Ayyar and Song, 2024; Ayyar et al., 2021; Li and Shah, 2019). A mechanistic model was developed in order to characterize the unknown drug-specific attributes for the *in vivo* CRISPR-Cas9 gene therapy (Ayyar and Song, 2024). The schematic diagram given in Supplementary Figure S1 represents the model structure. The model consists of one systemic compartment, which further includes a compartment to represent the mononuclear phagocyte system (MPS) (Kumar et al., 2023; Hoshyar et al., 2016) in which the drug will be internalized following a first-order rate constant of phagocytosis into the MPS ( $k_{\mathrm{int}}$ ) and degraded by $k_{\deg}$ . The LNP in the systemic circulation will be opsonized by plasma proteins to undergo bio-corona formation in the liver, represented by a $k_{\text{ass}}$ rate of association to the opsonins and a $k_{\text{dis}}$ rate of dissociation to the opsonins (Kumar et al., 2023; Monopoli et al., 2012). It is assumed that the LNP will interact with the LDL receptor via association $k_{\text{on},\text{LNP}}$ and dissociation $k_{\text{off},\text{LNP}}$ rate constants (Paunovska et al., 2022; Mager and Jusko, 2001; Johnson et al., 2014; Sebastiani et al., 2021). The unbound LNP degrades with a first-order rate constant $k_{\deg,\text{LNP}}$ . The complex releases the transgene product with $k_{\text{release}}$ (Miyazawa et al., 2024). The release Cas9 mRNA gets translated into Cas9 with $k_{\text{trans}}$ (Abdelhady et al., 2023; Miyazawa et al., 2024; Liu et al., 2024). The sgRNA and Cas9 form a ribonucleoprotein complex with $k_{\text{on},\text{RNP}}$ for association and $k_{\text{off},\text{RNP}}$ for dissociation (Abdelhady et al., 2023; Li et al., 2019; Rouet and Christ, 2019). The unbound sgRNA and Cas9 get degraded via $k_{\deg}$ (Fujiwara et al., 2016). In this model, the first-order rate of release $k_{release}$ for sgRNA and Cas9 mRNA is lumped.

**FIGURE 1:**
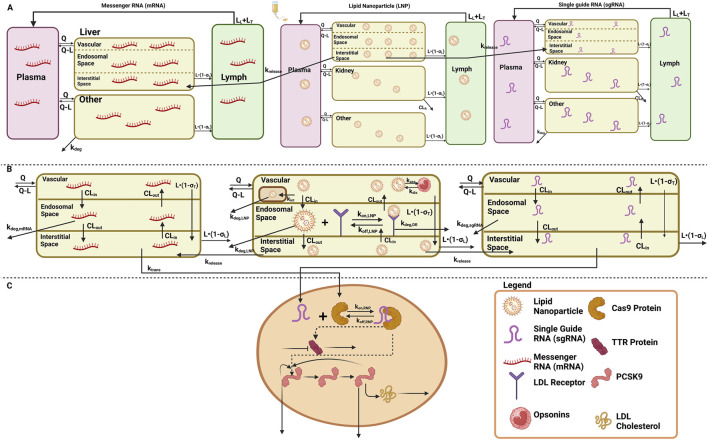
Schematic of the translational QSP model elucidating the disposition of three components, LNP, sgRNA, and mRNA, for *in vivo* CRISPR-Cas therapy. **(A)** A system PK model depicting the whole-body disposition for *in vivo* CRISPR-Cas therapy. The liver compartment within the model is divided into sub-compartments. **(B)** Organ-level structure of the system PK model for the disposition of all the components of *in vivo* CRISPR-Cas therapy, that is, LNP, sgRNA, and mRNA. **(C)** Cell-level system PD for *in vivo* CRISPR-Cas therapy.

The translational quantitative systems pharmacology model was developed to characterize the unique biodistribution of the three components associated with the therapy, namely, LNP, mRNA, and sgRNA (Shah and Betts, 2012; Betts et al., 2019; Ayyar et al., 2021). The model structure is shown in Figures 1A–C, and the series of Ordinary Differential Equations (ODEs) are provided in Supplementary Information S1. The model includes a liver compartment divided into vascular, interstitial, and cellular regions, and the kidney compartment is only present in the LNP and sgRNA, but it is absent in mRNA due to its higher molecular weight, which the mRNA will not eliminate via renal clearance (Meibohm and Zhou, 2012; Oladipo et al., 2022; Chen et al., 2022). The gene therapy enters the tissue vascular space via arterial plasma flow and exits via the venous plasma flow. Lymph flow is represented as 0.2% of the plasma flow. Once the LNP enters the vascular layer of the liver, it can get internalized into the MPS represented by $k_{\mathrm{int}}$ first-order rates of internalization and get degraded from the MPS represented by $k_{\text{degLNP}}$ . It can also undergo bio-corona formation via opsonins in the liver at a $k_{\text{ass}}$ rate of association to the opsonins and a $k_{\text{dis}}$ rate of dissociation to the opsonins. The LNP undergoes receptor-mediated endocytosis (Akinc et al., 2010; Miyazawa et al., 2024; Paunovska et al., 2022; Bisgaier et al., 1989; Johnson et al., 2014), which is represented by $CL_{\text{in}}$ , and exocytosis, which is represented by $CL_{\text{out}}$ . The mRNA and sgRNA undergo clathrin- or caveola-mediated endocytosis (Del Toro Runzer et al., 2023; den Roover and Aerts, 2023; Grimm et al., 2022), which is represented by $CL_{\text{in}}$ , and exocytosis, which is represented by $CL_{\text{out}}$ . $CL_{\text{in}}$ is defined as a product of the rate constant of endocytosis ( $k_{\text{in},\text{endo}}$ ) and volume of a particular compartment ( $V_{i}$ ), whereas $CL_{\text{out}}$ is defined as a product of the rate constant of exocytosis ( $k_{\text{out},\text{exo}}$ ) and physiological volume of a particular compartment ( $V_{i}$ ). In order to prevent parameter non-identifiability, the rate of endocytosis and exocytosis has been lumped for the LNP, mRNA, and sgRNA. The other route of uptake for the LNP, sgRNA, and mRNA is macropinocytosis via the paracellular pores using the convective lymph flow ( L ), where the reflection coefficient ( $\sigma_{V}$ ) represents the level of resistance provided to the delivery vehicle and to the transgene product by the vascular endothelial cells (Kumar et al., 2023; Shah and Betts, 2012). The LNPs, once in the endosomal layer, bind with the LDL receptor and get internalized in the interstitial layer. The LNP, sgRNA, and mRNA present in the interstitial space are taken up via pinocytosis by the endothelial cells. The delivery vehicle and transgene product exit through the convective lymph flow, where the level of resistance provided to the products via convection by the lymphatic openings is represented by the interstitial reflection coefficient. Once inside the interstitial layer, the LNP releases the transgene product with $k_{\text{release}}$ (Miyazawa et al., 2024). In the cell, the Cas9 mRNA gets translated into Cas9 with $k_{\text{trans}}$ . The Cas9 and sgRNA bind together to form an RNP complex to perform gene editing (Abdelhady et al., 2023).

According to the pharmacology and mechanism of action, the RNP complex cleaves the target DNA via indirect mechanisms by changing the DNA, causing a reduction in the protein level (Abdelhady et al., 2023). Therefore, the PD is modeled on a cellular layer via indirect response model-I, dependent on the concentration of the RNP in the liver as the TTR for the first case study (Ayyar et al., 2021; Mager and Jusko, 2008), and for the second case study, a feedback loop model (Friberg et al., 2002; Liu et al., 2022; Xia et al., 2021) was used based on the mechanism of action and pharmacology for PCSK9 as well as LDL cholesterol was measured in the blood samples the correction of biomarkers happened at the site of secretion, reduction in LDL-cholesterol is captured via precursor dependent model (Ayyar et al., 2021).

### Model development

#### Step 1: Development of the mechanistic model across species

A mechanistic model was developed to characterize unknown drug-specific attributes to understand unique PK/PD properties. The developed model, which is shown in Supplementary Figure S1, had a lot of drug-specific attributes, and data from all three species were modeled simultaneously to account for drug-specific attributes, which were then used for building a complex QSP model. Inter-individual variability (IIV) was turned on during this time to account for variability between species. The rate of association $\left(k_{\text{ass}}\right)$ and dissociation $\left(k_{\text{dis}}\right)$ with opsonins was estimated, along with the rate of dissociation between sgRNA and Cas9 $\left(k_{\text{off},\text{RNP}}\right)$ , the rate of translation from mRNA to Cas9 $\left(k_{\text{trans}}\right)$ , the rate of degradation for sgRNA $\left(k_{\deg,\text{sgRNA}}\right)$ , and the rate of degradation for the LNP-LDL complex $\left(k_{\deg,\text{DR}}\right)$ . The volume of one compartment was kept separate for mice and higher-order species (NHPs and humans) and was estimated. IIV was estimated on rate of association and dissociation to opsonins, rate of degradation of LNP-LDL complex and volume of one compartment for NHP and human. The residual error was estimated using proportional error model.

#### Step 2: Development of the translational QSP model in mice

A translational QSP model comprises a liver, kidney, and remainder (Figures 1A–C) for LNP and sgRNA, while for mRNA, the model comprises a liver and remainder. The dose was administered in the plasma compartment. The rate of endocytosis $\left(k_{\text{in},\text{endo}}\right)$ was fixed from NHPs and scaled using an allometric coefficient of −0.25 (Ayyar et al., 2021). The rate of exocytosis $\left(k_{\text{out},\text{exo}}\right)$ was estimated, and the rate of degradation for sgRNA $\left(k_{\deg,\text{sgRNA}}\right)$ was assumed to be the same as the rate of degradation for mRNA $\left(k_{\deg,\text{mRNA}}\right)$ , which was estimated. The rest of the drug-specific and species-specific parameters were fixed, as given in Table 2 and Supplementary Tables S1, S2. The residual standard error was estimated using a proportional error model.

**TABLE 2:** Parameter values for the translational QSP model in mice, NHPs, and humans.

Parameter	Description	Unit	Mice (28 g) (%RSE)	NHP (5 kg) (%RSE)	Human (71 kg) (%RSE)
$k_{\text{in},\text{endo}}$	Rate of endocytosis for LNP, sgRNA, and mRNA	1/h	0.14^a^	0.039 (43)	0.007 (1.44)
$k_{\text{out},\text{exo}}$	Rate of exocytosis for LNP, sgRNA, and mRNA	1/h	6.84 (13.4)	2,690 (65.4)	775 (0.602)
$k_{\deg,\text{DR}}$	Rate of degradation for the LNP-LDL complex	1/h	2.04^b^	2.04 (12)	5.15 (6.47)
${\text{LDL}}_{\text{tot}}$	LDL concentration	ug/mL	539^b^	539 (66.2)	84.5 (0.508)
$k_{\text{dis}}$	Rate of dissociation from opsonins	1/h	0.47^c^	8.64 (39)	0.186 (27.2)
$k_{\text{ass}}$	Rate of association from opsonins	1/h	1,550.34^c^	5.06 (14.5)	56.8 (16)
$k_{\text{release}}$	Rate of release of mRNA and sgRNA from LNPs	1/h	0.0056 (Miyazawa et al., 2024)	0.634 (5.31)	0.0056 (Miyazawa et al., 2024)
$k_{\deg,\text{LNP}}$	Rate of degradation of unbound LNP	1/h	1.6486 (Miyazawa et al., 2024)	1.6486 (Miyazawa et al., 2024)	0.101 (35.9)
$k_{\text{el}}$	Rate of elimination of the LDL receptor	1/h	0.231^d^ (Harwood and Pellarin, 1997)	0.231^d^ (Harwood and Pellarin, 1997)	0.009 (44.7)
$k_{\deg,\text{sgRNA}}$	Rate of degradation of sgRNA	1/h	0.378^e^	2.01^c^	2.01^c^
$k_{\deg,\text{mRNA}}$	Rate of degradation of mRNA	1/h	0.378 (46.5)	0.1232 (Miyazawa et al., 2024)	0.1232 (Miyazawa et al., 2024)
$k_{\text{on},\text{RNP}}$	Rate of association for the formation of the RNP complex	ug/mL/h	Calculated $k_{\text{on}}=\frac{\text{koff}}{K_{D}}$	Calculated $k_{\text{on}}=\frac{\text{koff}}{K_{D}}$	Calculated $k_{\text{on}}=\frac{\text{koff}}{K_{D}}$
$k_{\text{off},\text{RNP}}$	Rate of dissociation for the RNP complex	1/h	0.00188^c^	0.00188^c^	0.00188^c^
$K_{D}$	Equilibrium dissociation constant	nM	0.49 (Sternberg et al., 2014)	0.49 (Sternberg et al., 2014)	0.49 (Sternberg et al., 2014)
$k_{\mathrm{int}}$	Rate constant for phagocytosis into the mononuclear phagocytosis system	1/h	0.9063 (Miyazawa et al., 2024)	0.9063 (Miyazawa et al., 2024)	0.9063 (Miyazawa et al., 2024)
$k_{\text{on},\text{LNP}}$	Rate of association of the LNP-LDL complex	ug/mL/h	0.18 (Harwood and Pellarin, 1997)	0.18 (Harwood and Pellarin, 1997)	0.18 (Harwood and Pellarin, 1997)
$k_{\text{off},\text{LNP}}$	Rate of dissociation of the LNP-LDL complex	1/h	33.12 (Harwood and Pellarin, 1997)	33.12 (Harwood and Pellarin, 1997)	33.12 (Harwood and Pellarin, 1997)
$f_{u}$	Plasma free fraction of LNPs	—	0.002^f^ (Mager et al., 2012)	0.002^f^ (Mager et al., 2012)	0.002^f^ (Mager et al., 2012)
$k_{\text{trans}}$	Rate of translation from mRNA to Cas	1/h	0.36^c^	0.36^c^	0.36^c^
$f_{u}$	Plasma free fraction of RNA (sgRNA and mRNA)	—	0.15 (Ayyar et al., 2021)	0.15 (Ayyar et al., 2021)	0.15 (Ayyar et al., 2021)
${\text{TTR}}_{0}$	Baseline TTR concentration	%	—	100 (fixed)	100 (fixed)
$k_{\text{out},\text{TTR}}$	First-order rate constant for the degradation of the TTR protein	1/d	—	0.493 (15)	0.247 (17.5)
$I_{\max}$	Maximum inhibition for TTR	—	—	0.961 (0.625)	0.959 (6.55)
$IC_{50}$	RNP concentration at 50% of inhibition for TTR	ug/mL	—	4.77 (11.5)	0.3 (0.02)
γ	Gamma coefficient for TTR	—	—	0.31 (13.3)	—
$\text{PCSK}9_{0}$	Baseline PCSK9 in serum	%	—	100 (Fixed)	—
MTT	Mean transit time for PCSK9	day	—	14.5 (0.563)	—
$I_{\max}$	Maximum inhibition for PCSK9	—	—	0.771 (14.2)	—
$IC_{50}$	RNP concentration at 50% inhibition for PCSK9	ug/mL	—	21.5 (0.994)	—
γ	Gamma coefficient for PCSK9	—	—	1.1 (Fixed)	—
Γ	Gamma coefficient for LDL cholesterol	—	—	0.672 (18.5)	—
$k_{\deg,\text{LDL}}$	Rate of degradation for LDL cholesterol	1/d	—	4.66 (67.4)	—

TTR, transthyretin; PCSK9, proprotein convertase subtilisin/kexin type 9; LNP, lipid nanoparticle; mRNA, messenger mRNA; sgRNA, single guide RNA; LDL, low-density lipoprotein.

^a^ Fixed and scaled from NHPs at an allometric coefficient of −0.25.

^b^ Fixed and assumed the same as NHPs.

^c^ Fixed from the mechanistic model.

^d^ Calculated from the half-life of the LDL receptor (Harwood and Pellarin, 1997).

^e^ Assumed to be the same as for mRNA degradation.

^f^ Calculated from the renal clearance of mice (Mager et al., 2012).

#### Step 3: Development of the translational QSP model in NHPs.

The translational model was scaled to NHPs and was used to characterize the PK/PD properties of CRISPR-Cas9 therapy. The rate of endocytosis of the LNP, sgRNA, and mRNA was estimated $\left(k_{\text{in},\text{endo}}\right)$ . The rate of exocytosis of the LNP, sgRNA, and mRNA was estimated $\left(k_{\text{out},\text{exo}}\right)$ , along with the rate of degradation of the LNP-LDL complex $\left(k_{\text{degDR}}\right)$ , rate of association $\left(k_{\text{ass}}\right)$ and dissociation $\left(k_{\text{dis}}\right)$ with opsonins, and rate of release of the transgene product from the LNP $\left(k_{\text{release}}\right)$ . The concentration of the LDL receptor was also estimated. The residual standard error was estimated using a proportional error model. For the pharmacodynamics, the reduction in the TTR concentration was obtained after carrying out the modality with 1.5, 3, and 6 mg/kg of total RNA, and an indirect response model with inhibition at production was used (Mager and Jusko, 2008). The rate of degradation of the TTR protein $\left(k_{\text{out},\text{TTR}}\right)$ was estimated, along with the maximum inhibition effect $\left(I_{\max}\right)$ and the concentration at 50% of inhibition $\left(IC_{50}\right)$ . The gamma coefficient $\left(\gamma \right)$ was also estimated. The residual error was estimated using a proportional error model. The reduction in PCSK9 levels when the modality was dosed at 0.75 mg/kg and 1 mg/kg of total RNA was characterized using a feedback loop model based on the biology of the biomarker (Friberg et al., 2002). The mean transit time, maximum inhibition effect $\left(I_{\max}\right)$ , concentration at 50% of inhibition $\left(IC_{50}\right)$ , and gamma coefficient $\left(\gamma \right)$ were estimated. The residual error was estimated using a proportional error model. The reduction in LDL cholesterol levels was characterized using a precursor-dependent model (Ayyar et al., 2021), based on the biology of the biomarker of interest (Liu et al., 2022; Xia et al., 2021). The gamma coefficient $\left(\gamma \right)$ , as well as the rate of degradation of LDL cholesterol $\left(k_{\text{out},\text{LDL}}\right)$ , was estimated. The residual error was estimated using a proportional error model.

#### Step 4: Development of the translational QSP model in humans

The translational model was then scaled in humans where the plasma pharmacokinetics of LNPs was obtained after a short-term infusion of 2 h for doses of 0.1, 0.3, 0.7, and 1 mg/kg of total RNA. The rate of endocytosis for the LNP, sgRNA, and mRNA $\left(k_{\text{in},\text{endo}}\right)$ was estimated, as well as the rate of exocytosis $\left(k_{\text{out},\text{exo}}\right)$ and the rate of degradation for the LNP-LDL complex $\left(k_{\deg,\text{DR}}\right)$ . The rate of association $\left(k_{\text{ass}}\right)$ and dissociation $\left(k_{\text{dis}}\right)$ with opsonins was estimated. The rate of degradation of the unbound LNP $\left(k_{\deg,\text{LNP}}\right)$ was estimated as well. The concentration of LDL cholesterol and the rate of degradation for the LDL receptor $\left(k_{\text{el}}\right)$ were estimated as well. The residual error was estimated using a proportional error model. The reduction in TTR levels was characterized using an indirect response model with the inhibition for production of TTR proteins, which is the same as that used for NHPs. The rate of degradation of the TTR protein $\left(k_{\text{out},\text{TTR}}\right)$ , concentration at 50% of inhibition $\left(IC_{50}\right)$ , and maximum inhibition effect $\left(I_{\max}\right)$ were estimated. The residual error was estimated using a proportional error model.

### Model assumptions

Several key assumptions were made during the model building process: 1) single-pore disposition was assumed for sgRNA (Li and Shah, 2019; Baxter et al., 1994), 2) immediate release of the transgene product was assumed after administration (Miyazawa et al., 2024), 3) mRNA was assumed to be eliminated via exonuclease metabolism and tissue catabolism (Meibohm and Zhou, 2012), 4) negligible degradation of the RNP complex was assumed, and 5) assuming editing from the liver affects the reduction in biomarkers in the plasma as the liver is the target organ of interest for gene editing.

### Software

Data were extracted from the literature and represented as mean values using WebPlotDigitizer (Rohatgi, 2024). The analysis was performed in Monolix 2023R1 (Simulations Plus, 2023). The residual error model used per output that was implemented in the final model was

$${Var}_{i}={\left(\sigma_{slope}\cdot Y_{i}\right)}^{2}$$

Here, ${Var}_{i}$ is the variance of the ith observation, $Y_{i}$ is the ith model prediction, and $\sigma_{slope}$ represents the proportional variance, showing a linear relationship between the standard deviation and of the model output and $Y_{i}$ (Ayyar and Song, 2024; Simulations Plus, 2023). The model was reproduced to confirm for reproducibility using MATLAB R2022b (Simbiology) (Inc. TM, 2022).

### Global sensitivity analysis

A GSA was performed considering the complexity of the systems model (Marino et al., 2008; Alden et al., 2013). Sobol GSA was performed in Simbiology (Inc. TM, 2022) in different species in which drug-specific parameters were simultaneously perturbed with a sampling size of 1,000. The lower and upper bounds were different for each parameter and were based on the physiologically plausible value. SOBOL-based sensitivity indexes were simulated for 30 h. SOBOL indexes describe the importance of the parameter, along with its positive or negative correlation with the model output. The model output for mice was selected as the AUC of plasma PK for sgRNA and mRNA, the model output for NHPs was selected as the AUC of plasma PK for the LNP, and the model output for humans was selected as the AUC of plasma PK for LNPs.

## Results

### Modeling the tissue-level kinetics for unknown drug-specific attributes

A mechanistic model was built to characterize the tissue-level kinetics fitted to mouse, NHP, and human datasets simultaneously that included the measurements of the pharmacokinetics of the LNP, sgRNA, and mRNA. The model recapitulated the concentration of the delivery vehicle, as well as the mRNA and sgRNA in plasma, as shown in Supplementary Figure S2. The drug-specific attributes such as the rate of release of the transgene product from the complex $\left(k_{\text{release}}\right)$ , the rate of degradation of the unbound LNP $\left(k_{\deg,\text{LNP}}\right)$ , the rate of degradation of mRNA $\left(k_{\deg,\text{mRNA}}\right)$ , the rate of elimination of the LDL receptor $\left(k_{\text{el}}\right)$ , and the rate of synthesis of the LDL receptor $\left(k_{\text{syn}}\right)$ were calculated as a product of the total receptor concentration $\left({\text{LDL}}_{\text{tot}}\right)$ and the rate of elimination of the LDL receptor $\left(k_{\text{el}}\right)$ . The rate of association between sgRNA and Cas9 $\left(k_{\text{on},\text{RNP}}\right)$ , the rate of association of LNPs to the LDL receptor $\left(k_{\text{on},\text{LNP}}\right)$ , the rate of dissociation of LNPs to the LDL receptor $\left(k_{\text{off},\text{LNP}}\right)$ , the total LDL receptor concentration $\left({\text{LDL}}_{\text{tot}}\right)$ , and the rate of internalization of LNPs in the mononuclear phagocyte system $\left(k_{\mathrm{int}}\right)$ were fixed. The model estimated parameters included the rate of association for LNPs to opsonins $\left(k_{\text{ass}}\right)$ , which was estimated to be 1,550 1/h (14.9% RSE) with an IIV of 0.3334 (36.6% RSE). The rate of dissociation for LNPs to opsonins $\left(k_{\text{dis}}\right)$ was estimated to be 0.469 (5.43% RSE) with an IIV of 0.109 (39.3% RSE), the volume of plasma $\left(V_{\text{plasma}}\right)$ for NHPs and humans was estimated to be 3.01 mL (64.8% RSE) with an IIV 1.71 (26.9% RSE), the volume of plasma for mice was 0.718 mL (0.304% RSE), and the rate of dissociation between sgRNA and Cas9 $\left(k_{\text{off},\text{RNP}}\right)$ was estimated to be 0.001 1/h (17.5% RSE). The rate of translation from mRNA to Cas9 $\left(k_{\text{trans}}\right)$ was estimated to be 0.364 1/h (11.9% RSE), the rate of degradation for sgRNA $\left(k_{\deg,\text{sgRNA}}\right)$ was estimated to be 2.01 1/h (5.11% RSE), and the rate of degradation for the LNP-LDL complex $\left(k_{\deg,\text{DR}}\right)$ was estimated to be 3.73 (12.7% RSE) with an IIV of 0.257 (39.4% RSE). All the fixed and estimated drug-specific attributes are given in Supplementary Table S3.

### QSP modeling in mice: a case study with investigational gene therapy

**FIGURE 2:**
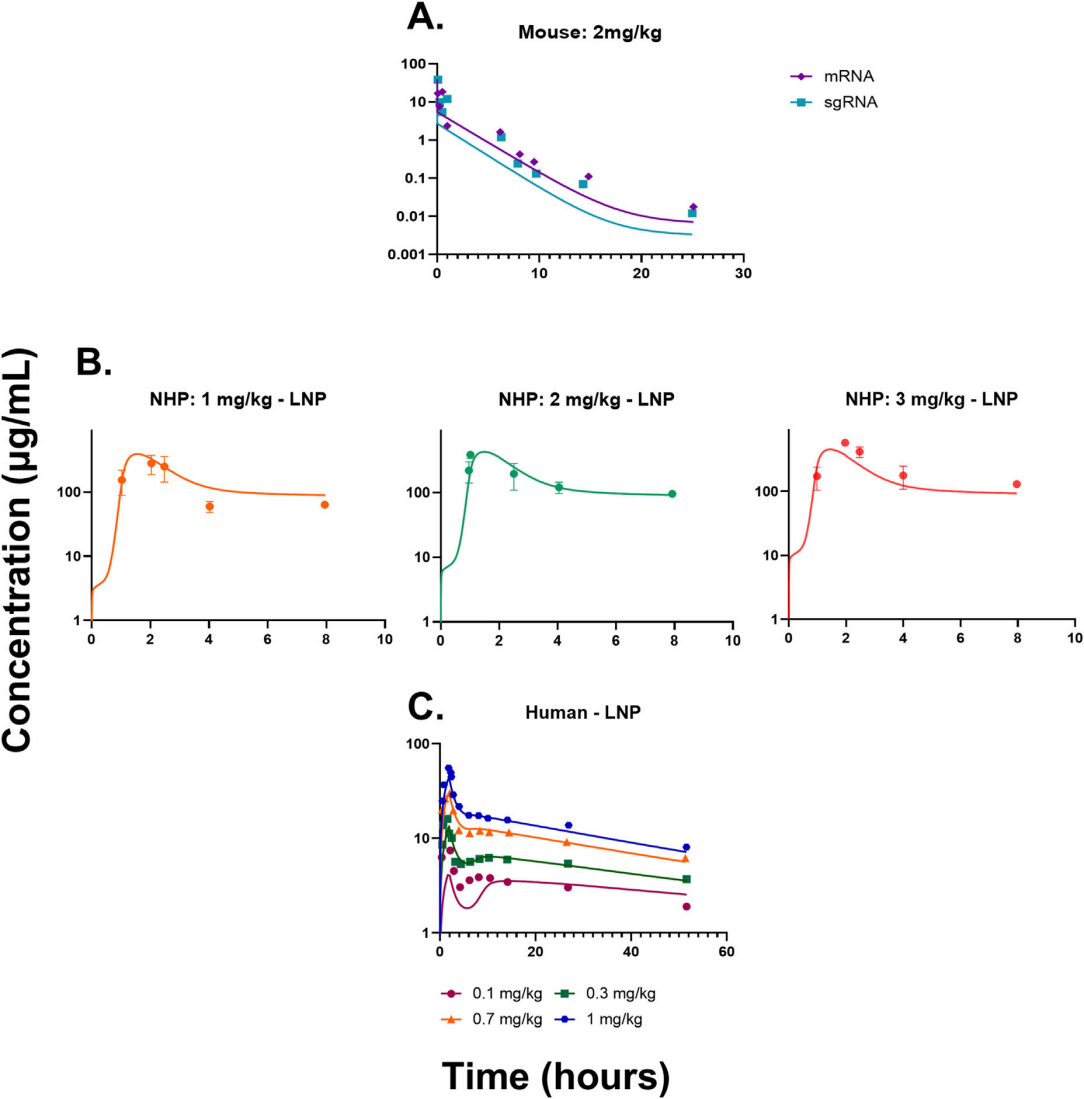
**(A)** Model fittings for sgRNA and mRNA in plasma for mice from a dose of 2 mg/kg of total RNA dosed via IV bolus. **(B)** Model fittings for LNPs in plasma for NHPs for doses of 1, 2, and 3 mg/kg of IV infusion for 2 h. **(C)** Model fittings for LNPs in plasma for humans for doses of 0.1, 0.3, 0.7, and 1 mg/kg of IV infusion for 2 h. The points are the observed data obtained from the literature (Abdelhady et al., 2023; Finn et al., 2018; Gillmore et al., 2021), and the lines are the model fittings.

The QSP model (Figures 1A–C and Supplementary Figure S1) was fitted to the mouse pharmacokinetics data, for sgRNA and mRNA in plasma (Finn et al., 2018). The model characterized the PK profile for a dose of 2 mg/kg of total RNA (Figure 2A). The drug-specific attribute such as the rate of exocytosis of the LNP, sgRNA, and mRNA $\left(k_{\text{out},\text{exo}}\right)$ was estimated to be 6.84 1/h (13.4% RSE). Other drug-specific and species-specific attributes, which have been assumed to be similar to those in NHPs, are the rate of degradation for the LNP-LDL complex $\left(k_{\deg,\text{DR}}\right)$ and LDL concentration $\left({\text{LDL}}_{\text{tot}}\right)$ . Drug-specific attributes such as the rate of association to opsonins $\left(k_{\text{ass}}\right)$ , the rate of dissociation to opsonins $\left(k_{\text{dis}}\right)$ , the rate of dissociation to RNPs $\left(k_{\text{off},\text{RNP}}\right)$ , and the rate of translation from mRNA to Cas $\left(k_{\text{trans}}\right)$ were fixed from the mechanistic model, whereas the rate of degradation of mRNA $\left(k_{\deg,\text{mRNA}}\right)$ was estimated to be 0.378 (46.5% RSE), and the rate of degradation of sgRNA ( $\left.k_{\deg,sgRNA}\right)$ was assumed to be similar to the rate of degradation of mRNA due to similar faster degradation in plasma for sgRNA and mRNA. The rest of the drug-specific and species-specific attributes were fixed (Supplementary Tables S1, S2; Table 2).

### Translational QSP modeling in NHPs: a case study with NTLA-2001 and VERVE-101

**FIGURE 3:**
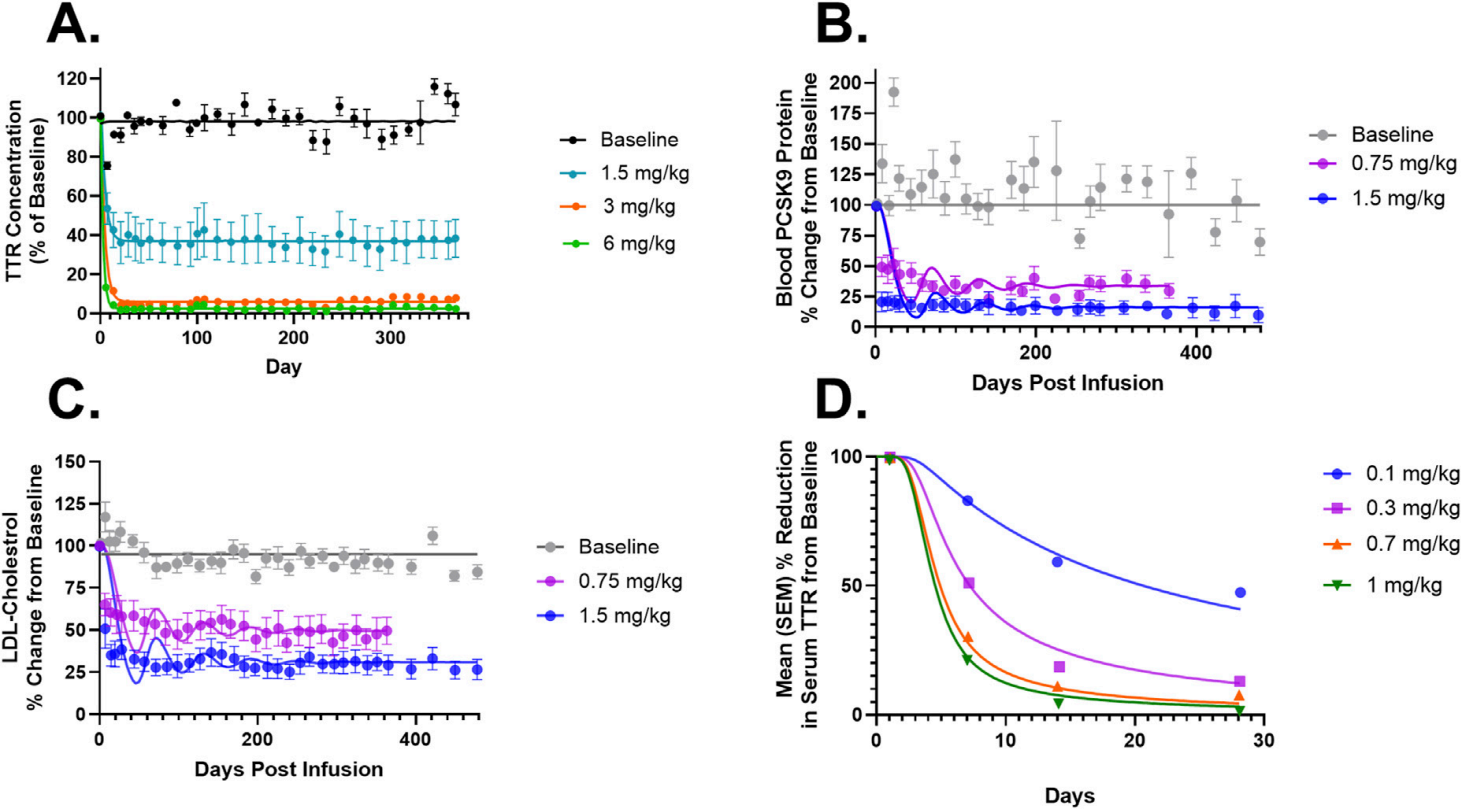
**(A)** Model fittings for serum TTR proteins in NHPs for doses of 1.5, 3, and 6 mg/kg via IV infusion for 2 h. **(B)** Model fittings for serum PCSK9 levels in NHPs for doses of 0.75 and 1.5 mg/kg via IV infusion for 2 h. **(C)** Model fittings for serum LDL cholesterol levels in NHPs for doses of 0.75 and 1.5 mg/kg via IV infusion for 2 h. **(D)** Model fittings for serum TTR proteins in humans for doses of 0.1, 0.3, 0.7, and 1 mg/kg via IV infusion for 2 h. The points are the observed data obtained from the literature (Gillmore et al., 2021; Lee et al., 2023; Gane et al., 2022).

The QSP model was then scaled to NHPs using its respective physiological flows and volumes, which are provided in Supplementary Tables S1, S2. The model recapitulated the dose-dependent changes in PK (Figure 2B) (Gillmore et al., 2021). The drug-specific attributes such as the rate of endocytosis for the LNP, sgRNA, and mRNA $\left(k_{\text{in},\text{endo}}\right)$ was estimated to be 0.039 1/h (43% RSE); the rate of exocytosis $\left(k_{\text{out},\text{exo}}\right)$ for LNP, sgRNA, and mRNA was estimated to be 2,690 1/h (65.4% RSE); the rate of degradation for the LNP-LDL complex $\left(k_{\deg,\text{DR}}\right)$ was estimated to be 2.04 1/h (12% RSE); the total LDL receptor concentration $\left({\text{LDL}}_{\text{tot}}\right)$ was estimated to be 539 ug/mL (66.2% RSE); the rate of dissociation for LNPs from opsonins $\left(k_{\text{dis}}\right)$ was estimated to be 8.64 1/h (39% RSE); the rate of association for LNPs from opsonins $\left(k_{\text{ass}}\right)$ was estimated to be 5.06 1/h (14.5% RSE); and the rate of release of the transgene product from LNPs $\left(k_{\text{release}}\right)$ was estimated to be 0.634 1/h (5.31% RSE). Drug-specific attributes such as the rate of degradation of sgRNA $\left(k_{\deg,\text{sgRNA}}\right)$ , the rate of dissociation to RNPs $\left(k_{\text{off},\text{RNP}}\right)$ , and the rate of translation from mRNA to Cas were fixed from the mechanistic model $\left(k_{\text{trans}}\right)$ . The rest of the drug-specific attributes were fixed, as given in Table 2. The pharmacodynamic responses were characterized from two different drugs for the first case study, which is used for calibration. The PD of NTLA-2001 was characterized using the indirect response model with inhibition at production of the TTR protein. The rate of degradation of the TTR protein $\left(k_{\text{out},\text{TTR}}\right)$ was estimated to be 0.493 1/d (15% RSE), the maximum inhibition effect for the TTR protein $\left(I_{\max}\right)$ was estimated to be 0.961 (0.625% RSE), the concentration at 50% of inhibition $\left({\text{IC}}_{50}\right)$ was estimated to be 4.77 ug/mL (11.5% RSE), and the gamma coefficient $\left(\gamma \right)$ was estimated to be 0.31 (13.3% RSE). In the second case study, which is used for validation, the PD of VERVE-101 was used to characterize the reduction in PCSK9 levels, leading to the reduction in LDL cholesterol levels. Here, the feedback loop model was used for estimating the reduction in PCSK9 levels. The mean transit time for PCSK9 $\left(\text{MTT}\right)$ was estimated to be 14.5 days (0.563% RSE), the maximum inhibition for PCSK9 $\left(I_{\max}\right)$ was estimated to be 0.771 (14.2% RSE), and $IC_{50}$ of the RNP complex was estimated to be 21.5 ug/mL (0.994% RSE). The reduction in LDL cholesterol levels was characterized using a precursor-dependent model, where the gamma coefficient $\left(\gamma \right)$ was estimated to be 0.672 (18.5% RSE) and the rate of degradation of LDL cholesterol $\left(k_{\deg,\text{LDL}}\right)$ levels was estimated to be 4.66 1/d (67.4% RSE). The model well characterized the responses for both the drugs and explained their mechanism. In the case of the reduction in TTR proteins, the model predicted the rapid decline in the PD of the drug, whereas in the case of PCSK9 and LDL cholesterol levels, due to the lack of PK data from VERVE-101, the model could not capture the rapid decrease in PCSK9 and LDL cholesterol levels (Figures 3A–C).

### Translational QSP modeling in humans: a case study with NTLA-2001

The QSP model was scaled to humans using physiological flows and volumes, as shown in Supplementary Tables S1, S2. The resulting model characterized the dose-dependent changes in the PK of LNPs, and it modestly under-predicted the lowest dose (Figure 2C). The drug-specific attributes such as the rate of endocytosis for the LNP, sgRNA, and mRNA $\left(k_{\text{in},\text{endo}}\right)$ was estimated to be 0.007 1/h (1.44% RSE); the rate of exocytosis for the LNP, sgRNA, and mRNA $\left(k_{\text{out},\text{exo}}\right)$ was estimated to be 775 1/h (0.602% RSE); the rate of degradation for the LNP-LDL complex $\left(k_{\deg,\text{DR}}\right)$ was estimated to be 5.15 1/h (6.47 % RSE); the total LDL receptor concentration $\left({\text{LDL}}_{\text{tot}}\right)$ was estimated to be 84.5 ug/mL (0.508% RSE); the rate of dissociation for LNPs from opsonins $\left(k_{\text{dis}}\right)$ was estimated to be 0.186 1/h (27.2% RSE); the rate of association for LNPs from opsonins $\left(k_{\text{ass}}\right)$ was estimated to be 56.8 1/h (16% RSE); the rate of degradation for unbound LNPs $\left(k_{\deg,\text{LNP}}\right)$ was estimated to be 0.101 1/h (35.9% RSE); and the rate of degradation for the LDL receptor $\left(k_{\text{el}}\right)$ was estimated to be 0.009 1/h (44.7% RSE). The final estimates from the mechanistic model were used for parameters such as the rate of dissociation to RNPs $\left(k_{\text{off},\text{RNP}}\right)$ , the rate of degradation of sgRNA $\left(k_{\deg,\text{sgRNA}}\right)$ , and the rate of translation from mRNA to Cas, which were fixed from the mechanistic model $\left(k_{\text{trans}}\right)$ . The rest of the drug-specific parameters were fixed and are given in Table 2. The pharmacodynamic responses for NTLA-2001 targeting TTR proteins were characterized using the indirect response model with inhibition at production of the TTR protein (Figure 3D). The rate of degradation for TTR proteins $\left(k_{\deg,\text{TTR}}\right)$ was estimated to be 0.247 1/d (17.5% RSE), $IC_{50}$ was estimated to be 0.3 ug/mL (0.02% RSE), and $I_{\max}$ was estimated to be 0.959 (6.55% RSE).

### Monte Carlo simulations for the QSP model

**FIGURE 4:**
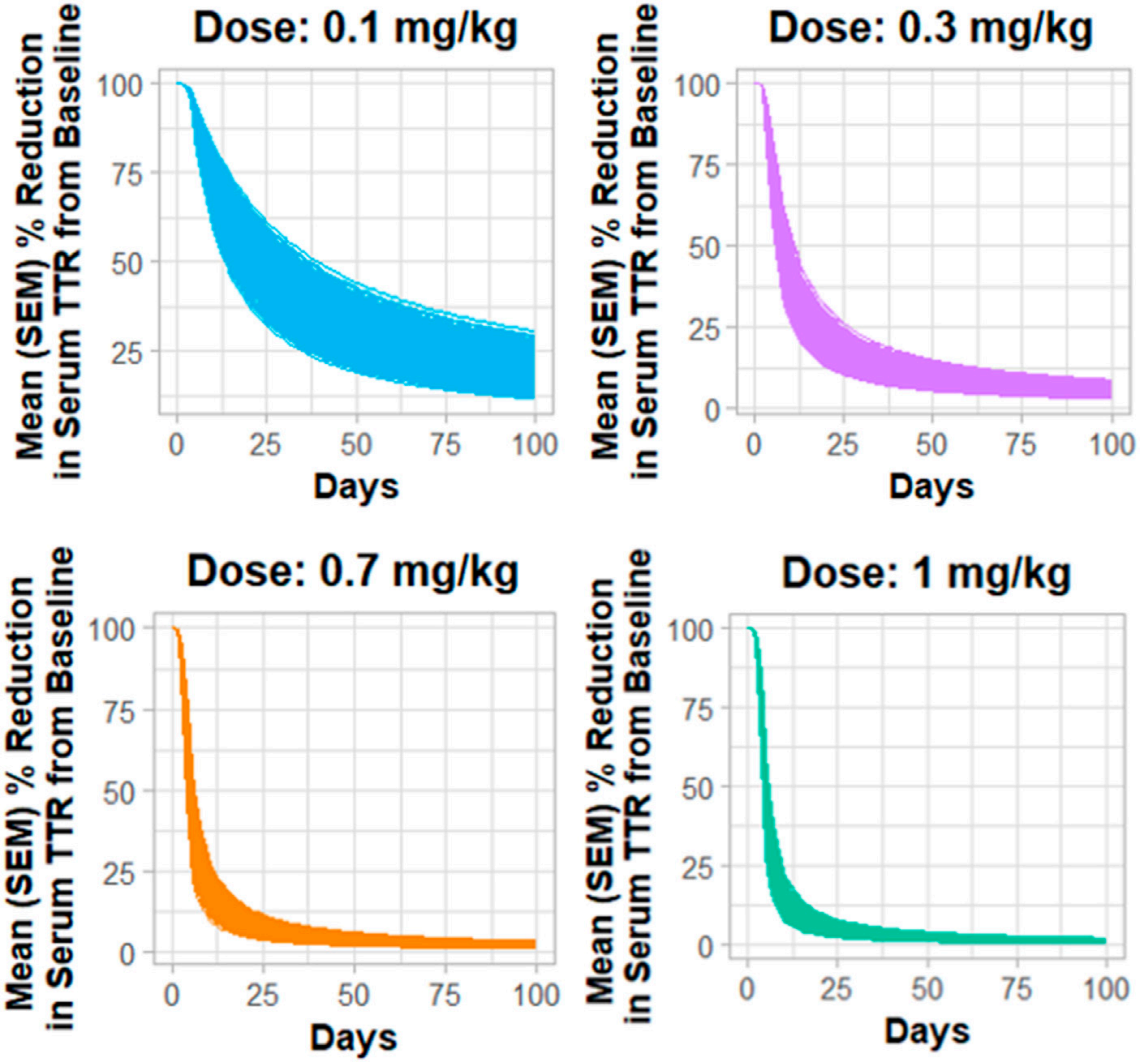
Monte Carlo simulations for serum TTR proteins in humans assuming for 20% variability for the assessment of the dose–response relationship.

Figure 4 describes the Monte Carlo simulations (Singh et al., 2021) for the percentage change from baseline of change in serum TTR protein after the administration of *in vivo* CRISPR-Cas9 gene therapy. Monte Carlo simulations were performed for 1,000 subjects, assuming 20% variability between the subjects. The simulations revealed that a lower dose of 0.1 mg/kg of total RNA administered exhibited responses in fewer patients, whereas the subsequent dose level of 0.3 mg/kg exhibited a higher response with fewer chances of relapse. The other two higher doses (0.7 mg/kg and 1 mg/kg) of the total RNA administered showed a higher depth of response.

### Model predictive assessment

**FIGURE 5:**
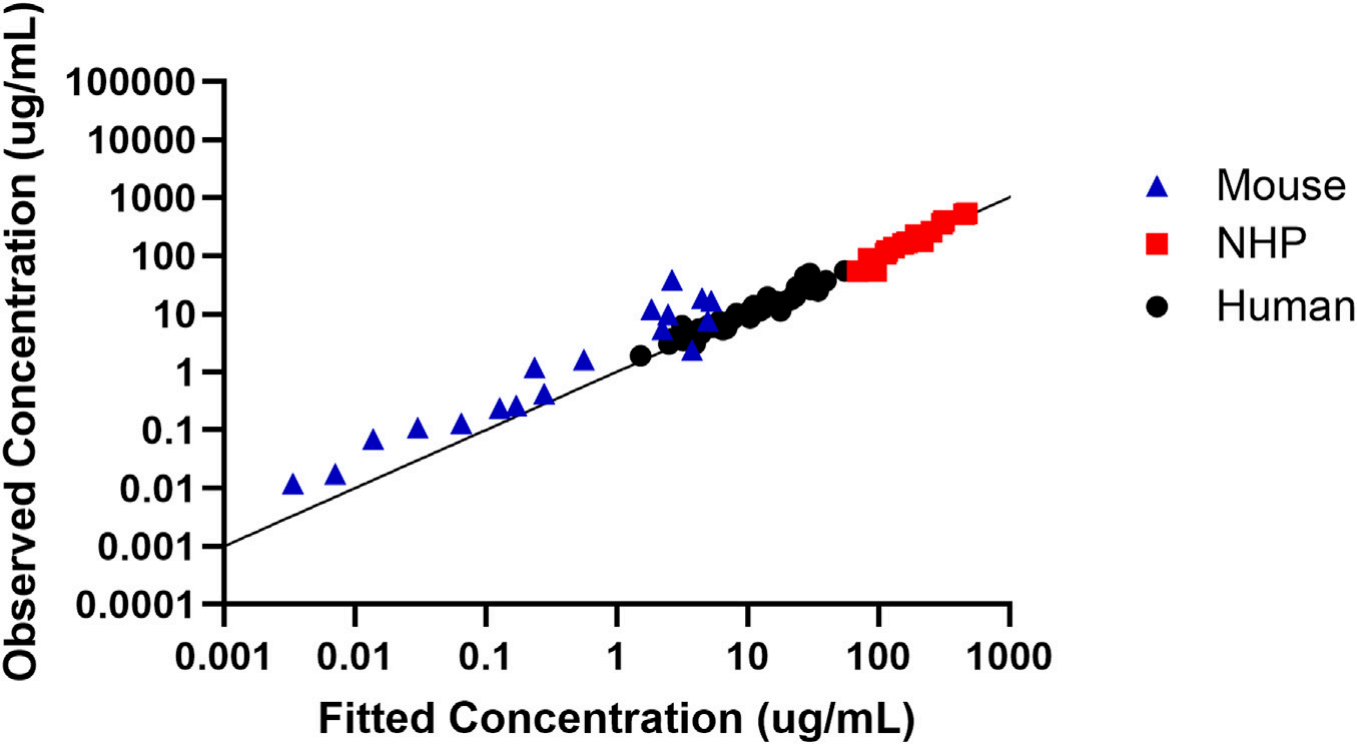
Assessment of the model predictive performance in multiple species. Observed *versus* fitted concentration of *in vivo* CRISPR-Cas therapy in multiple species with the line of identity.

In order to assess the predictive performance of the built translational QSP model, Figure 5 shows that the model adequately predicts all the *in vivo* CRISPR disposition in multiple species, as indicated by the line of identity.

### Global sensitivity analysis

**FIGURE 6:**
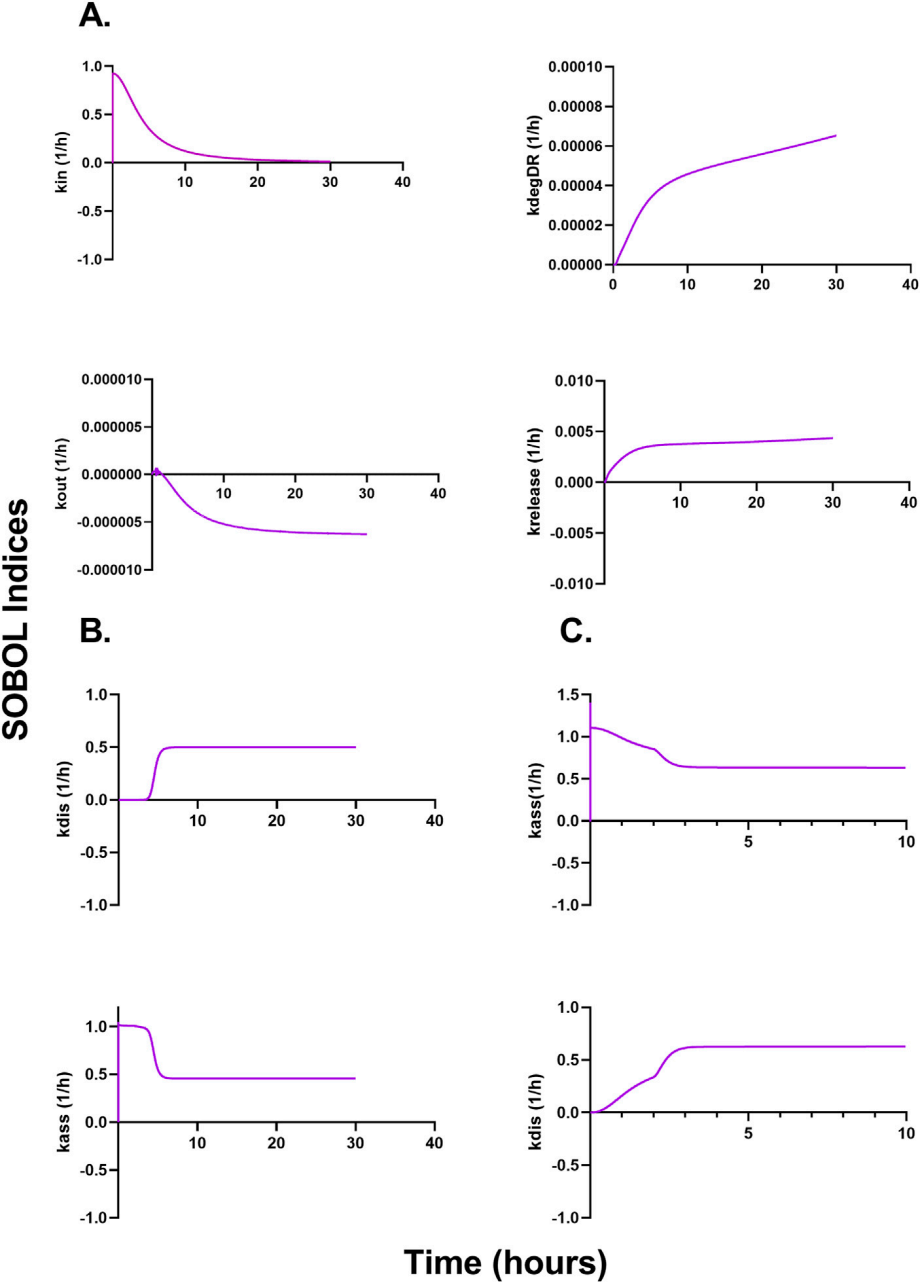
Global sensitivity analysis (GSA) in multiple species accessing the impact of parameters on the model output according to SOBOL indexes. **(A)** Outcomes of GSA on mice. **(B)** Outcomes of GSA on NHPs. **(C)** Outcomes of GSA on humans.

Figures 6A–C describe a time-variant GSA (SOBOL) on the QSP model developed for NTLA-2001 to characterize the impact on plasma profiles. For mouse sgRNA and mRNA plasma profiles, the rate of endocytosis for LNP, sgRNA, and mRNA, as well as the rate of exocytosis of LNP, sgRNA and mRNA, was found to be sensitive. The rate of degradation of the LNP-LDL complex, as well as the rate of release of the transgene product, was found to be influential on the SOBOL indexes. For NHPs, the rate of dissociation for LNPs to opsonins, as well as the rate of association for LNPs to opsonins, was found to be sensitive on the SOBOL indexes. For humans, the rate of association for LNPs to opsonins and the rate of dissociation for LNPs to opsonins were found to be sensitive in SOBOL indexes.

## Discussion

Due to their high selectivity for targeted gene editing, CRISPR-Cas-based therapies, as well as other oligonucleotide therapeutics, have gained significant traction across industries and academia over the past decade. CRISPR-Cas therapies offer an ability to edit genes selectively and correct the protein production to cure diseases. Currently, there are five *in vivo* CRISPR-Cas therapies under development (Abdelhady et al., 2023). The quantitative impact of the putative determinants of CRISPR-Cas activity is only partially understood. It has unique PK-PD characteristics compared to small molecule- and protein-based therapeutics, and it is challenging to establish PK-PD relationships for CRISPR-Cas. For example, there are no established paradigms to leverage preclinical data to predict the safe dose for CRISPR-Cas in humans. In gene therapies, there are previously published studies in oligonucleotides for siRNA, which include a mechanistic platform mPBPK-PD model developed for GaINAc-siRNA, which translates the information from preclinical species to humans (Ayyar and Song, 2024; Ayyar et al., 2021; Zhang et al., 2020). There are previously published models for LNPs, which are present in single species; however, they do not translate the results to clinical settings (Kumar et al., 2023; Mager et al., 2012; Dong et al., 2015; Rajoli et al., 2015; Henrique et al., 2017; Gilkey et al., 2015; MacCalman et al., 2009). An mPBPK/QSP translational model which describes the tissue disposition and protein expression dynamics of LNP-mRNA was found. The study also predicted the efficacious dose via a translational model and included virtual patient population to determine the dosing schedule and efficacious dose (Miyazawa et al., 2024). This model has applications for vaccines and mRNA therapeutics, but this model cannot cover the disposition of CRIPSR-Cas9 and its components, such as sgRNA, as well as the formation of the ribonucleoprotein complex to exert its effect.

In this paper, we developed a novel QSP platform model to characterize the biodistribution and mechanism of action of *in vivo* CRISPR-Cas9 therapy. The model recapitulated the PK of preclinical species such as mice and NHPs, as well as was translated to humans. The model was built with the data obtained from the literature (Abdelhady et al., 2023; Miyazawa et al., 2024; Finn et al., 2018; Gillmore et al., 2021; Lee et al., 2023; Gane et al., 2022), which include the measurements of sgRNA and mRNA in plasma of mice, and LNPs in plasma of NHPs and humans for characterization of disposition. There are measurements in serum plasma for TTR proteins, as well as for PCSK9 and LDL cholesterol levels, for a different *in vivo* CRISPR-Cas therapy. The biodistribution model includes receptor-mediated endocytosis of LNPs, the internalization in the mononuclear phagocyte system, bio-corona formation via binding with opsonins in plasma, the release of sgRNA and mRNA, tissue catabolism, and renal clearance. Briefly, once the LNP enters the vascular space, the drug undergoes opsonization by plasma proteins, which leads to bio-corona formation, or the LNP will escape the vascular space by undergoing phagocytosis by MPS cells. These cells are responsible for the distribution and clearance of LNPs. This rate of internalization was fixed to a reported value (Miyazawa et al., 2024). The rate constant for receptor-mediated endocytosis for LNP-, clathrin-, or caveola-mediated endocytosis for sgRNA and mRNA was fixed in mice by allometry scaling from NHPs, whereas it was estimated in NHPs as 0.039 1/h and humans as 0.007 1/h. This value indicated that the value of uptake in the liver cells decreases across species, and further experiments are needed to confirm the change. The rate of exocytosis was estimated to be 6.84 1/h in mice, 2,690 1/h in NHPs, and 775 1/h in humans. The association with opsonins was estimated in NHPs and humans to be 5.06 1/h and 56.8 1/h, respectively, indicating that the LNP undergoes higher opsonization across species, whereas the rate of dissociation with opsonins was estimated to be 8.64 1/h in NHP and 0.186 1/h in humans, respectively, indicating that the LNP has a decreased amount of dissociation with opsonins across species. The total LDL receptor concentration across species decreases from 539 ug/mL estimated in NHPs to 84.5 ug/mL, being a possible reason for the lower uptake of LNP.

To summarize, a translational QSP model was successfully developed to characterize the preclinical-to-clinical translation of *in vivo* CRISPR-Cas therapies. The model characterized the mechanism of action of two different drugs, NTLA-2001 and VERVE-101, in addition to characterizing the complex biodistribution. Processes such as exocytosis of the LNPs and dissociation of LNPs from opsonins characterized a redistribution phase in the LNP exposure. The model also simulated the variability in response using Monte Carlo simulations. This QSP platform can be applied in the translation of *in vivo* CRISPR-Cas gene therapies, can be used to gather information regarding the first-in-human dose for these therapies, and provides us with a better understanding of the dose–exposure–response relationship for this modality.

Following the generation of key mechanistic data, future modifications can be done to the model by accounting for changes in physiochemical properties (charge and pI) of the delivery vehicle, as well as accounting for the structural asymmetry of mRNA and sgRNA. A future modification can account for the complex biodistribution of AAV, which is not well explained as a delivery vehicle for CRISPR-Cas therapies. This model can be further expanded based on mRNA and sgRNA PK data in all the organs of the body to characterize the whole-body biodistribution for this modality. Experiments for the characterization of fluid-phase macropinocytosis for mRNA and sgRNA can help leverage the information about the uptake for the transgene product, similar to the experiments used for the characterization of antibodies (Haigler et al., 1979). Furthermore, a drug–trial–disease model can be linked with a toxicokinetic model to characterize the toxicity, as well as help predict the first-in-human dose. The utility of this QSP model will include translating the preclinical PK and PD to humans and help guide the development and translation of *in vivo* CRISPR-Cas therapies.

## Conclusion

In summary, the approach taken in this work demonstrated that several basic, well-established processes that govern pharmacokinetics, such as species physiology, binding, transport, and target-mediated drug disposition, as well as pharmacodynamics such as mechanism of action, biomarkers, and turnover processes, can be assembled to build a translational QSP model for novel CRISPR-Cas therapies. The proposed framework will promote the mechanistic and quantitative reasoning to guide experimental designs during the preclinical and early clinical development of CRISPR-Cas therapies. This systems model will form the basis of characterizing the PK-PD properties of CRISPR-Cas therapeutics.

## Data Availability

The original contributions presented in the study are included in the article/Supplementary Material; further inquiries can be directed to the corresponding authors.
