# Supplementary material for: A quantitative systems pharmacology (QSP) platform for preclinical to clinical translation of in-vivo CRISPR-Cas therapy
Source: Front Pharmacol. 2024 Sep 20;15:1454785. doi: 10.3389/fphar.2024.1454785 (PMC11449743; doi:10.3389/fphar.2024.1454785)
Supplement: Supplementary file 1 [file Table1.DOCX]

**A quantitative systems pharmacology (QSP) platform for preclinical to clinical translation of in-vivo CRISPR-Cas therapy**

**Supplementary Information**

**Devam A. Desai ^[1]^, Stephan Schmidt ^[1]^ and Rodrigo Cristofoletti ^[1]^**

**^[1]^ Center of Pharmacometrics and Systems Pharmacology, University of Florida, Orlando, FL, US**

**Supplementary Figure (F) 1.** Schematic of mechanistic model for in-vivo CRISPR Cas therapy for identification of drug specific attributes

**
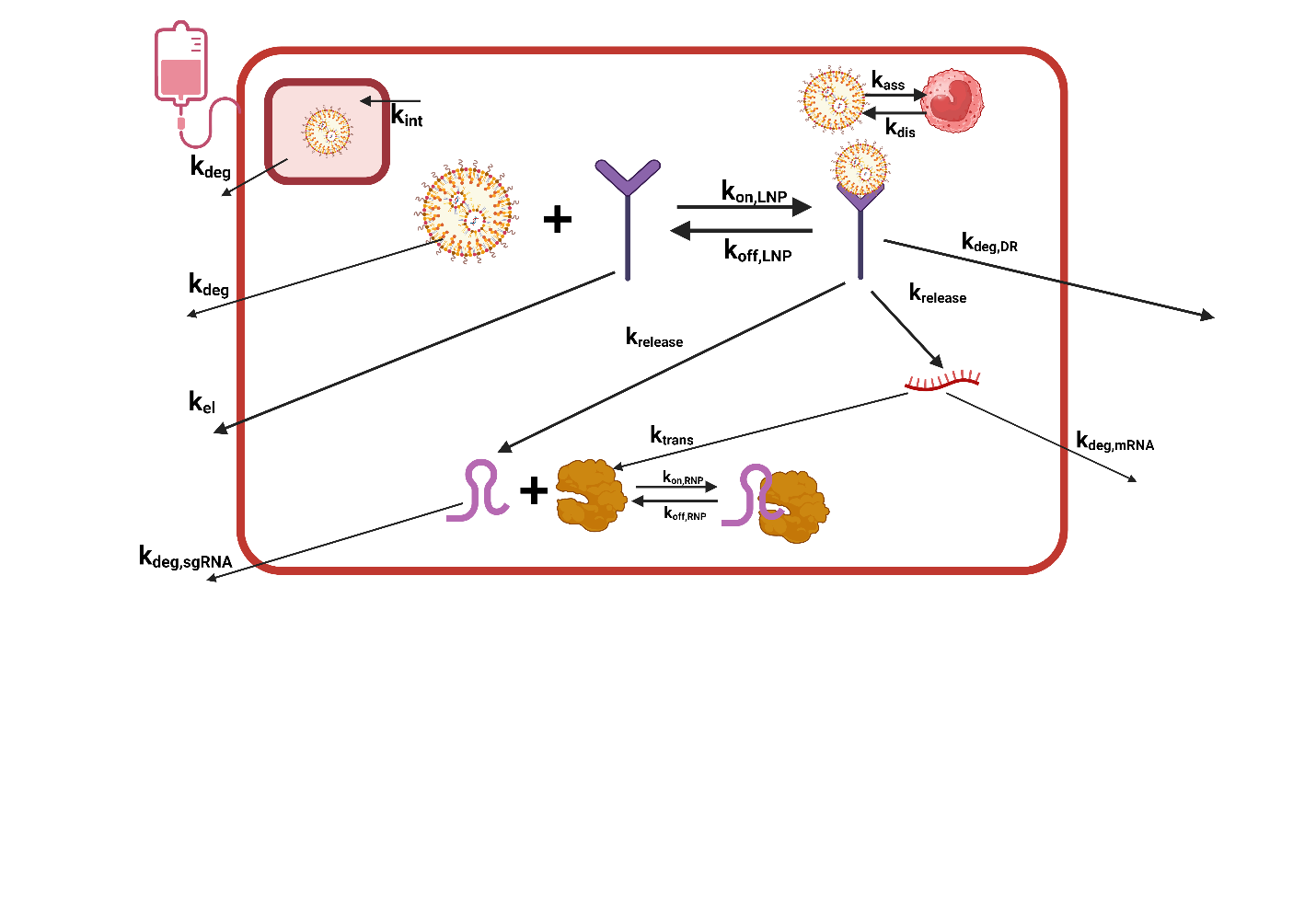
**

**Supplementary Figure (F) 2.**

Model assessment for mechanistic model for in-vivo CRISPR Cas therapy, majority of the data is near the line of identity indicative of the fact that the model was able to well characterize the drug-specific attributes of the mechanistic model for in-vivo CRISPR Cas therapy.

**
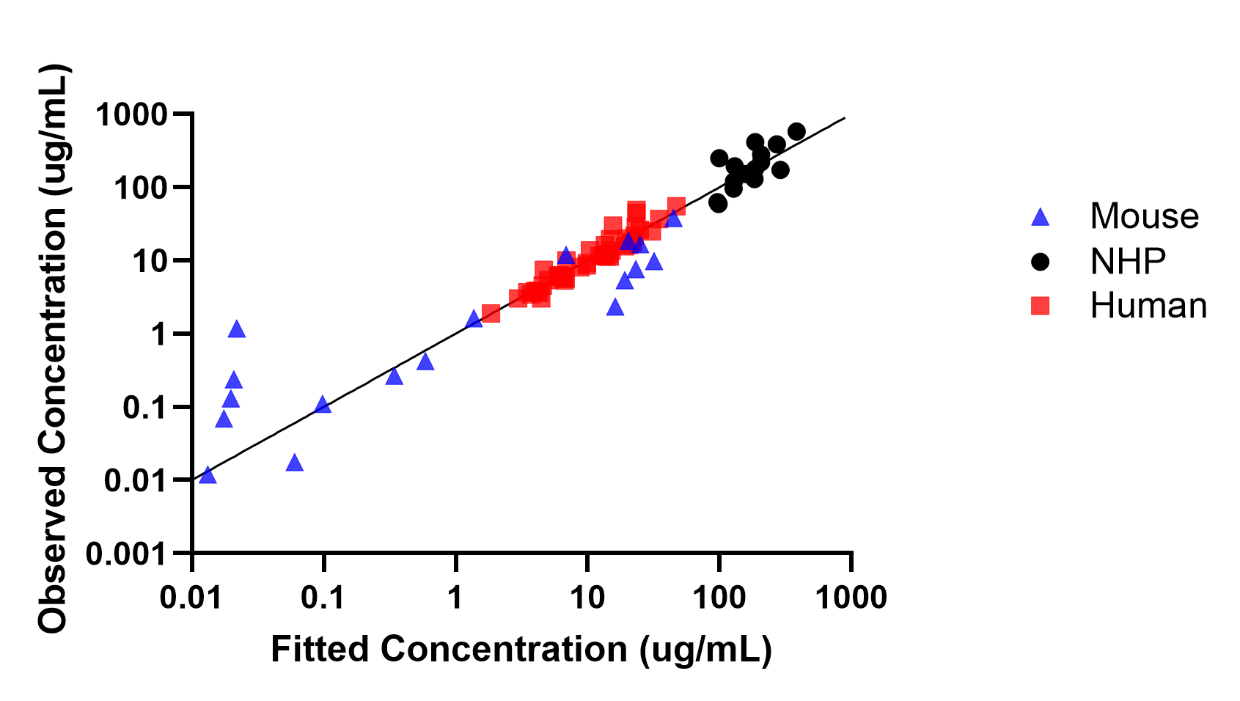
**

**Supplementary Table (T) 1.** Physiological volumes used in mouse, NHP and humans.

| Parameter | Description | Unit | Mouse (28g) | | NHP  (5kg) | Human  (71kg) |
| --- | --- | --- | --- | --- | --- | --- |
| $\mathbf{V}_{\mathbf{plasma}}$ | Plasma volume in circulation | mL | | 0.94 | 187 | 3126 |
| $\mathbf{V}_{\mathbf{lymph}}$ | Volume of lymph | mL | | 0.113 | 25.1 | 274 |
| $\mathbf{V}_{\mathbf{livervas}}$ | Plasma volume in liver vasculature | mL | | 0.164 | 15.9 | 183 |
| $\mathbf{V}_{\mathbf{liverendo}}$ | Endosomal volume of liver | mL | | 0.009 | 0.934 | 10.7 |
| $\mathbf{V}_{\mathbf{liverinter}}$ | Cellular and Interstitial volume of liver | mL | | 1.62 | 157.4 | 429 |
| $\mathbf{V}_{\mathbf{MPS}}$ | Volume of mononuclear phagocytotic system | mL | | 0.123^a^ | 12 ^a^ | 137.1 ^a^ |
| $\mathbf{V}_{\mathbf{K}}$ | Plasma, Interstitial and Cellular volume of kidney | mL | | 0.47 | 25.9 | 332 |
| $\mathbf{V}_{\mathbf{rem}}$ | Extracellular volume of remainder tissue | mL | | 5.1 | 1087 | 12394 |

^a^ Value is calculated from (1)

All the volumes are adapted from (2-4)

**Supplementary Table (T) 2.** Physiological flows used in mouse, NHP and humans

| Parameter | Description | Unit | Mouse  (28g) | NHP  (5kg) | Human  (71kg) |
| --- | --- | --- | --- | --- | --- |
| $\mathbf{Q}_{\mathbf{L}}$ | Plasma flow to liver | mL/h | 100 | 1251 | 13210 |
| $\mathbf{L}_{\mathbf{L}}$ | Lymph flow to liver | mL/h | 0.2 | 2.502 | 26.42 |
| $\mathbf{Q}_{\mathbf{T}}$ | Plasma flow to remainder tissue | mL/h | 204 | 10358 | 97677 |
| $\mathbf{L}_{\mathbf{T}}$ | Lymph flow to remainder tissue | mL/h | 0.46 | 20.716 | 195.354 |
| $\mathbf{Q}_{\mathbf{K}}$ | Plasma flow to kidney | mL/h | 68.5 | 3237 | 36402 |
| $\mathbf{L}_{\mathbf{K}}$ | Lymph flow to kidney | mL/h | 0.137 | 6.464 | 72.804 |
| $\boldsymbol{\sigma}_{\boldsymbol{L}}$ | Reflection coefficient | Dimensionless | 0.2 | 0.2 | 0.2 |
| $\boldsymbol{\sigma}_{\mathbf{V}}$ | Reflection coefficient (pinocytosis) | Dimensionless | 0.9 | 0.9 | 0.9 |
| $\mathbf{GFR}$ | Glomerular filtration rate | mL/h | 13.8 | 1138 | 7200 |

All the species-specific attributes are fixed from (2-5)

**Supplementary Table (T) 3.** Final estimates for all the parameter in the mechanistic model fitted simultaneously to mouse, NHP and human where IIV accounts for inter-species variability.

| Parameter | Description | Unit | Estimate (%RSE) | IIV (%RSE) |
| --- | --- | --- | --- | --- |
| $\mathbf{K}_{\mathbf{D}}$ | Equilibrium dissociation constant | nM | 0.088 ^(6)^ | - |
| $\mathbf{k}_{\mathbf{on,RNP}}$ | Association rate constant for RNP | ug/mL/hr | $k_{on,RNP}= \frac{k_{off,RNP}}{K_{D}}$ | - |
| $\mathbf{k}_{\mathbf{release}}$ | Rate of release of mRNA and sgRNA from LNP | 1/h | 0.0056 ^(1)^ | - |
| $\mathbf{k}_{\mathbf{int}}$ | Rate of internalization for phagocytosis in MPS | 1/h | 0.9063 ^(1)^ | - |
| $\mathbf{k}_{\mathbf{deg}}$ | Rate of degradation for unbound LNP | 1/h | 1.6486 ^(1)^ | - |
| $\mathbf{k}_{\mathbf{deg,mRNA}}$ | Rate of degradation of mRNA | 1/h | 0.1232 ^(1)^ | - |
| $\mathbf{k}_{\mathbf{el}}$ | Rate of elimination for LDL receptor | 1/h | 0.231^a^ ^(7)^ | - |
| $\mathbf{LD}\mathbf{L}_{\mathbf{tot}}$ | Total LDL receptor concentration | ug/mL | 5000^b (7)^ | - |
| $\mathbf{k}_{\mathbf{on,LNP}}$ | Rate of association LNP-LDL | ug/mL/hr | 0.18^(7)^ | - |
| $\mathbf{k}_{\mathbf{off,LNP}}$ | Rate of dissociation LNP-LDL | 1/h | 33.12^(7)^ | - |
| $\mathbf{k}_{\mathbf{ass}}$ | Rate of association to opsonins | 1/h | 1550 (14.9) | 0.3334 (36.6) |
| $\mathbf{k}_{\mathbf{dis}}$ | Rate of dissociation to opsonins | 1/h | 0.469 (5.43) | 0.109 (39.3) |
| $\mathbf{k}_{\mathbf{off,RNP}}$ | Rate of dissociation to RNP | 1/h | 0.001 (17.5) | - |
| $\mathbf{k}_{\mathbf{trans}}$ | Rate of translation from mRNA to Cas protein | 1/h | 0.364 (11.9) | - |
| $\mathbf{k}_{\mathbf{deg,sgRNA}}$ | Rate of degradation for sgRNA | 1/h | 2.01 (5.11) | - |
| $\mathbf{k}_{\mathbf{deg,DR}}$ | Rate of degradation for LNP-LDL complex | 1/h | 3.73 (12.7) | 0.257 (39.4) |
| $\mathbf{V}_{\mathbf{plasma}}$ | Volume of plasma for NHP and Human | mL | 3.01 (64.8) | 1.71 (26.9) |
| $\mathbf{V}_{\mathbf{plasma}}$ | Volume of plasma for mouse | mL | 0.718 (0.304) | - |

**^a^** Calculated from half life of LDL receptor (7)

^b^ Fixed from (7) in-vitro assay assumed to be same across species

$$k_{\mathrm{syn}}=\mathrm{LDL}_{\mathrm{tot}}\cdot k_{\mathrm{el}}$$

**S1. Series of Differential equations (DEQ) for Translational QSP model:**

**Lipid Nanoparticle (LNP):**

$$\frac{\mathrm{dC}_{\mathrm{LNPPlasma}}}{\mathrm{dt}}=\frac{\left( Q_{L}-L_{L} \right)\cdot C_{\mathrm{LNPlivervas}}+\left( Q_{T}-L_{T} \right)\cdot C_{\mathrm{LNPrem}}+\left( Q_{K}-L_{K} \right)\cdot C_{\mathrm{LNPKidney}}+\left( L_{L}+L_{T}+L_{K} \right)\cdot C_{\mathrm{LNPlymph}}-Q_{L}\cdot C_{\mathrm{LNPPlasma}}-Q_{T}\cdot C_{\mathrm{LNPPlasma}}-Q_{K}\cdot C_{\mathrm{LNPPlasma}}}{V_{\mathrm{plasma}}}$$

$$\frac{dC_{\mathrm{LNPlymph}}}{\mathrm{dt}}=\frac{L_{L}\cdot\left( 1-\sigma_{L} \right)\cdot C_{\mathrm{LNPliverinter}}+L_{T}\cdot\left( 1-\sigma_{L} \right)\cdot C_{\mathrm{LNPrem}}+L_{K}\cdot\left( 1-\sigma_{L} \right)\cdot C_{\mathrm{LNPKidney}}-\left( L_{L}+L_{K}+L_{T} \right)\cdot C_{\mathrm{LNPlymph}}}{V_{\mathrm{lymph}}}$$

$$\frac{dC_{\mathrm{LNPlivervas}}}{\mathrm{dt}}=\frac{\left( \begin{aligned} Q_{L}\cdot C_{\mathrm{LNPPlasma}}-\left( Q_{L}-L_{L} \right)\cdot C_{\mathrm{LNPlivervas}}-CL_{\mathrm{inlivervas}}\cdot C_{\mathrm{LNPlivervas}}-L_{L}\cdot\left( 1-\sigma_{V} \right)\cdot C_{\mathrm{LNPlivervas}}-\left( k_{\mathrm{int}}\cdot V_{\mathrm{livervas}}\cdot C_{\mathrm{LNPlivervas}} \right) \\ -\left( k_{\mathrm{ass}}\cdot V_{\mathrm{livervas}}\cdot C_{\mathrm{LNPlivervas}} \right)+\left( k_{\mathrm{dis}}\cdot V_{\mathrm{opsions}}\cdot C_{\mathrm{Opsions}} \right)+CL_{out,DR}\cdot C_{\mathrm{LNPLDLcomplex}} \end{aligned} \right)}{V_{\mathrm{livervas}}}$$

$$\frac{dC_{\mathrm{LNPMPS}}}{\mathrm{dt}}=\frac{k_{\mathrm{int}}\cdot V_{\mathrm{livervas}}\cdot C_{\mathrm{LNPlivervas}}-k_{deg,LNP}\cdot V_{\mathrm{MPS}}\cdot C_{\mathrm{LNPMPS}}}{V_{\mathrm{MPS}}}$$

$$\frac{dC_{\mathrm{Opsions}}}{\mathrm{dt}}=\frac{\left( k_{\mathrm{ass}}\cdot V_{\mathrm{livervas}}\cdot C_{\mathrm{LNPlivervas}}-k_{\mathrm{dis}}\cdot V_{\mathrm{opsions}}\cdot C_{\mathrm{Opsions}} \right)}{V_{\mathrm{opsions}}}$$

$$\frac{dC_{\mathrm{LNPliverendo}}}{\mathrm{dt}}=\frac{\begin{aligned} CL_{\mathrm{inlivervas}}\cdot C_{\mathrm{LNPlivervas}}-\left( k_{deg,LNP}\cdot C_{\mathrm{LNPliverendo}}\cdot V_{\mathrm{liverendo}} \right)+\left( k_{off,LNP}\cdot V_{\mathrm{liverendo}}\cdot C_{\mathrm{LNPLDLcomplex}} \right) \\ -\left( k_{on,LNP}\cdot C_{\mathrm{LNPliverendo}}\cdot C_{\mathrm{LDL}}\cdot V_{\mathrm{liverendo}} \right)+CL_{\mathrm{inliverinter}}\cdot C_{\mathrm{LNPliverinter}} \end{aligned}}{V_{\mathrm{liverendo}}}$$

$$\frac{dC_{\mathrm{LDL}}}{\mathrm{dt}}=\frac{k_{off,LNP}\cdot V_{\mathrm{liverendo}}\cdot C_{\mathrm{LNPLDLcomplex}}-k_{on,LNP}\cdot C_{\mathrm{LNPliverendo}}\cdot C_{\mathrm{LDL}}\cdot V_{\mathrm{liverendo}}-k_{\mathrm{el}}\cdot V_{\mathrm{liverendo}}\cdot C_{\mathrm{LDL}}+k_{\mathrm{syn}}\cdot V_{\mathrm{liverendo}}+CL_{\mathrm{inDR}}\cdot C_{\mathrm{LNPLDLcomplex}}}{V_{\mathrm{liverendo}}}$$

$$\frac{dC_{\mathrm{LNPLDLcomplex}}}{\mathrm{dt}}=\frac{k_{on,LNP}\cdot C_{\mathrm{LNPliverendo}}\cdot C_{\mathrm{LDL}}\cdot V_{\mathrm{liverendo}}-k_{off,LNP}\cdot V_{\mathrm{liverendo}}\cdot C_{\mathrm{LNPLDLcomplex}}-CL_{\mathrm{inDR}}\cdot C_{\mathrm{LNPLDLcomplex}}-k_{deg,DR}\cdot V_{\mathrm{liverendo}}\cdot C_{\mathrm{LNPLDLcomplex}}}{V_{\mathrm{liverendo}}}$$

$$\frac{dC_{\mathrm{LNPliverinter}}}{\mathrm{dt}}= \frac{\begin{aligned} CL_{\mathrm{outDR}}\cdot C_{\mathrm{LNPLDLcomplex}}-CL_{\mathrm{inliverinter}}\cdot C_{\mathrm{LNPliverinter}}+L_{L}\cdot\left( 1-\sigma_{V} \right)\cdot C_{\mathrm{LNPlivervas}}-\left( k_{\mathrm{release}}\cdot C_{\mathrm{LNPliverinter}}\cdot V_{\mathrm{liverinter}} \right) \\ -\left( k_{\mathrm{release}}\cdot C_{\mathrm{LNPliverinter}}\cdot V_{\mathrm{liverinter}} \right)-L_{L}\cdot\left( 1-\sigma_{L} \right)\cdot C_{\mathrm{LNPliverinter}} \end{aligned}}{V_{\mathrm{liverinter}}}$$

$$\frac{dC_{\mathrm{LNPKidney}}}{\mathrm{dt}}=\frac{Q_{K}\cdot C_{\mathrm{LNPPlasma}}-\left( Q_{K}-L_{K} \right)\cdot C_{\mathrm{LNPKidney}}-CL_{R}\cdot C_{\mathrm{LNPKidney}}-L_{K}\cdot\left( 1-\sigma_{L} \right)\cdot C_{\mathrm{LNPKidney}}}{V_{\mathrm{Kidney}}}$$

$$\frac{dC_{\mathrm{LNPrem}}}{\mathrm{dt}}=\frac{\left( Q_{T}\cdot C_{\mathrm{LNPPlasma}}-\left( Q_{T}-L_{T} \right)\cdot C_{\mathrm{LNPrem}}-k_{deg,LNP}\cdot V_{\mathrm{rem}}\cdot C_{\mathrm{LNPrem}}-L_{T}\cdot\left( 1-\sigma_{L} \right)\cdot C_{\mathrm{LNPrem}} \right)}{V_{\mathrm{rem}}}$$

**Single guide RNA (sgRNA):**

$$\frac{dC_{\mathrm{sgRNAPlasma}}}{\mathrm{dt}}=\frac{\begin{aligned} \left( Q_{L}-L_{L} \right)\cdot C_{\mathrm{sgRNAlivervas}}+\left( Q_{T}-L_{T} \right)\cdot C_{\mathrm{sgRNArem}}+\left( Q_{K}-L_{K} \right)\cdot C_{\mathrm{sgRNAkideny}}+\left( L_{L}+L_{T}+L_{K} \right)\cdot C_{\mathrm{sgRNAlymph}}-Q_{L}\cdot C_{\mathrm{sgRNAPlasma}} \\ -Q_{T}\cdot C_{\mathrm{sgRNAPlasma}}-Q_{K}\cdot C_{\mathrm{sgRNAPlasma}} \end{aligned}}{V_{\mathrm{Plasma}}}$$

$$\frac{dC_{\mathrm{sgRNAlymph}}}{\mathrm{dt}}=\frac{L_{L}\cdot\left( 1-\sigma_{L} \right)\cdot C_{\mathrm{sgRNAliverinter}}+L_{T}\cdot\left( 1-\sigma_{L} \right)\cdot C_{\mathrm{sgRNArem}}-\left( L_{L}+L_{T}+L_{K} \right)\cdot C_{\mathrm{sgRNAlymph}}+L_{K}\cdot\left( 1-\sigma_{L} \right)\cdot C_{\mathrm{sgRNAkidnry}}}{V_{\mathrm{lymph}}}$$

$$\frac{dC_{\mathrm{sgRNAlivervas}}}{\mathrm{dt}}=\frac{Q_{L}\cdot C_{\mathrm{sgRNAPlasma}}-\left( Q_{L}-L_{L} \right)\cdot C_{\mathrm{sgRNAlivervas}}-CL_{\mathrm{inlivervas}}\cdot C_{\mathrm{sgRNAlivervas}}+CL_{\mathrm{outliverendo}}\cdot C_{\mathrm{sgRNAliverendo}}-L_{L}\cdot\left( 1-\sigma_{V} \right)\cdot C_{\mathrm{sgRNAlivervas}}}{V_{\mathrm{livervas}}}$$

$$\frac{dC_{\mathrm{sgRNAliverendo}}}{\mathrm{dt}}=\frac{\begin{aligned} CL_{\mathrm{inlivervas}}\cdot C_{\mathrm{sgRNAlivervas}}-CL_{\mathrm{outliverendo}}\cdot C_{\mathrm{sgRNAliverendo}}-\left( k_{deg,sgRNA}\cdot C_{\mathrm{sgRNAliverendo}}\cdot V_{\mathrm{liverendo}} \right)+CL_{\mathrm{inliverinter}}\cdot C_{\mathrm{sgRNAliverinter}} \\ -CL_{\mathrm{outliverendo}}\cdot C_{\mathrm{sgRNAliverendo}} \end{aligned}}{V_{\mathrm{liverendo}}}$$

$\frac{dC_{\mathrm{sgRNAliverinter}}}{\mathrm{dt}}=\frac{\begin{aligned} CL_{\mathrm{outliverendo}}\cdot C_{\mathrm{sgRNAliverendo}}-CL_{\mathrm{inliverinter}}\cdot C_{\mathrm{sgRNAliverinter}}+L_{L}\cdot\left( 1-\sigma_{V} \right)\cdot C_{\mathrm{sgRNAlivervas}}+\left( k_{\mathrm{release}}\cdot C_{\mathrm{LNPliverinter}}\cdot V_{\mathrm{liverinter}} \right) \\ -L_{L}\cdot\left( 1-\sigma_{L} \right)\cdot C_{\mathrm{sgRNAliverinter}}-\left( k_{\mathrm{release}}\cdot V_{\mathrm{liverinter}}\cdot C_{\mathrm{sgRNAliverinter}} \right) \end{aligned}}{V_{\mathrm{liverinter}}}$

$$\frac{dC_{\mathrm{sgRNAkidney}}}{\mathrm{dt}}=\frac{Q_{K}\cdot C_{\mathrm{sgRNAPlasma}}-\left( Q_{K}-L_{K} \right)\cdot C_{\mathrm{sgRNAkidney}}-CL_{\mathrm{RRNA}}\cdot C_{\mathrm{sgRNAkidney}}-L_{K}\cdot\left( 1-\sigma_{L} \right)\cdot C_{\mathrm{sgRNAkidney}}}{V_{\mathrm{kidney}}}$$

$$\frac{dC_{\mathrm{sgRNArem}}}{\mathrm{dt}}=\frac{Q_{T}\cdot C_{\mathrm{sgRNAPlasma}}-\left( Q_{T}-L_{T} \right)\cdot C_{\mathrm{sgRNArem}}-k_{deg,sgRNA}\cdot V_{\mathrm{rem}}\cdot C_{\mathrm{sgRNArem}}-L_{T}\cdot\left( 1-\sigma_{L} \right)\cdot C_{\mathrm{sgRNArem}}}{V_{\mathrm{rem}}}$$

**Messenger RNA (mRNA) and Cas protein:**

$$\frac{dC_{\mathrm{mRNAPlasma}}}{\mathrm{dt}}=\frac{\begin{aligned} \left( Q_{L}-L_{L} \right)\cdot C_{\mathrm{mRNAlivervas}}+\left( Q_{T}-L_{T} \right)\cdot C_{\mathrm{mRNArem}}+\left( L_{L}+L_{T} \right)\cdot C_{\mathrm{mRNAlymph}}-Q_{L}\cdot C_{\mathrm{mRNAPlasma}} \\ -Q_{T}\cdot C_{\mathrm{mRNAPlasma}} \end{aligned}}{V_{\mathrm{Plasma}}}$$

$$\frac{dC_{\mathrm{mRNAlymph}}}{\mathrm{dt}}=\frac{L_{L}\cdot\left( 1-\sigma_{L} \right)\cdot C_{\mathrm{mRNAliverinter}}+L_{T}\cdot\left( 1-\sigma_{L} \right)\cdot C_{\mathrm{mRNArem}}-\left( L_{L}+L_{T} \right)\cdot C_{\mathrm{mRNAlymph}}}{V_{\mathrm{lymph}}}$$

$$\frac{dC_{\mathrm{mRNAlivervas}}}{\mathrm{dt}}=\frac{Q_{L}\cdot C_{\mathrm{mRNAPlasma}}-\left( Q_{L}-L_{L} \right)\cdot C_{\mathrm{mRNAlivervas}}-CL_{\mathrm{inlivervas}}\cdot C_{\mathrm{mRNAlivervas}}+CL_{\mathrm{outliverendo}}\cdot C_{\mathrm{mRNAliverendo}}-L_{L}\cdot\left( 1-\sigma_{V} \right)\cdot C_{\mathrm{mRNAlivervas}}}{V_{\mathrm{livervas}}}$$

$$\frac{dC_{\mathrm{mRNAliverendo}}}{\mathrm{dt}}=\frac{\begin{aligned} CL_{\mathrm{inlivervas}}\cdot C_{\mathrm{mRNAlivervas}}-CL_{\mathrm{outliverendo}}\cdot C_{\mathrm{mRNAliverendo}}-\left( k_{deg,mRNA}\cdot C_{\mathrm{mRNAliverendo}}\cdot V_{\mathrm{liverendo}} \right)+CL_{\mathrm{inliverinter}}\cdot C_{\mathrm{mRNAliverinter}} \\ -CL_{\mathrm{outliverendo}}\cdot C_{\mathrm{mRNAliverendo}} \end{aligned}}{V_{\mathrm{liverendo}}}$$

$$\frac{dC_{\mathrm{mRNAliverinter}}}{\mathrm{dt}}=\frac{\begin{aligned} CL_{\mathrm{outliverendo}}\cdot C_{\mathrm{mRNAliverendo}}-CL_{\mathrm{inliverinter}}\cdot C_{\mathrm{mRNAliverinter}}+L_{L}\cdot\left( 1-\sigma_{V} \right)\cdot C_{\mathrm{mRNAlivervas}}+\left( k_{\mathrm{release}}\cdot C_{\mathrm{LNPliverinter}}\cdot V_{\mathrm{liverinter}} \right) \\ -L_{L}\cdot\left( 1-\sigma_{L} \right)\cdot C_{\mathrm{mRNAliverinter}}-\left( k_{\mathrm{trans}}\cdot V_{\mathrm{liverinter}}\cdot C_{\mathrm{mRNAliverinter}} \right) \end{aligned}}{V_{\mathrm{liverinter}}}$$

$$\frac{dC_{\mathrm{mRNArem}}}{\mathrm{dt}}=\frac{Q_{T}\cdot C_{\mathrm{mRNAPlasma}}-\left( Q_{T}-L_{T} \right)\cdot C_{\mathrm{mRNArem}}-k_{deg,mRNA}\cdot V_{\mathrm{rem}}\cdot C_{\mathrm{mRNArem}}-L_{T}\cdot\left( 1-\sigma_{L} \right)\cdot C_{\mathrm{mRNArem}}}{V_{\mathrm{rem}}}$$

**Ribonucleoprotein Complex (RNP):**

$$\frac{dC_{\mathrm{sgRNAcell}}}{\mathrm{dt}}=\frac{\left( k_{\mathrm{release}}\cdot V_{\mathrm{liverinter}}\cdot C_{\mathrm{sgRNAliverinter}} \right)-k_{on,RNP}\cdot C_{\mathrm{sgRNAcell}}\cdot C_{\mathrm{Proteincas}}\cdot V_{\mathrm{liverinter}}+\left( k_{off,RNP}\cdot V_{\mathrm{liverinter}}\cdot C_{\mathrm{RNP}} \right)}{V_{\mathrm{liverinter}}}$$

$$\frac{dC_{\mathrm{Proteincas}}}{\mathrm{dt}} =\frac{\left( k_{\mathrm{trans}}\cdot V_{\mathrm{liverinter}}\cdot C_{\mathrm{mRNAliverinter}} \right)+\left( k_{off,RNP}\cdot V_{\mathrm{liverinter}}\cdot C_{\mathrm{RNP}} \right)-\left( k_{on,RNP}\cdot C_{\mathrm{sgRNAcell}}\cdot C_{\mathrm{Proteincas}}\cdot V_{\mathrm{liverinter}} \right)}{V_{\mathrm{liverinter}}}$$

$$\frac{dC_{\mathrm{RNP}}}{\mathrm{dt}}=\frac{k_{on,RNP}\cdot C_{\mathrm{sgRNAcell}}\cdot C_{\mathrm{Proteincas}}\cdot V_{\mathrm{liverinter}}-\left( k_{off,RNP}\cdot V_{\mathrm{liverinter}}\cdot C_{\mathrm{RNP}} \right)}{V_{\mathrm{liverinter}}}$$

**Drug Specific attributes:**

$$CL_{\mathrm{inDR}}=k_{in,endo}\cdot V_{\mathrm{liverendo}}$$

$$CL_{\mathrm{inlivervas}}=k_{in,endo}\cdot V_{\mathrm{livervas}}$$

$$CL_{\mathrm{outDR}}=k_{out,exo}\cdot V_{\mathrm{liverendo}}$$

$$CL_{\mathrm{inliverinter}}=k_{in,endo}\cdot V_{\mathrm{liverinter}}$$

$$CL_{\mathrm{outliverinter}}=k_{out,exo}\cdot V_{\mathrm{liverendo}}$$

$$k_{\mathrm{syn}}=\mathrm{LDL}_{\mathrm{tot}}\cdot k_{\mathrm{el}}$$

$$CL_{R}=f_{u}\cdot GFR$$

**Reduction of TTR proteins:**

$$k_{in,TTR}=k_{out,TTR}\cdot TTR_{0}$$

$$\frac{\mathrm{dTTR}}{\mathrm{dt}}=k_{in,TTR}\cdot\left( 1-\left( I_{\max}\cdot\frac{C_{\mathrm{RNP}}}{IC_{50}+C_{\mathrm{RNP}}} \right) \right)-k_{out,TTR}\cdot TTR ;TTR_{0}=100$$

**Reduction of PCSK9 and LDL cholesterol:**

$$k_{\mathrm{tr}}=\left( \frac{n+1}{\mathrm{MTT}} \right)$$

$$\frac{\mathrm{dProl}}{\mathrm{dt}}=k_{\mathrm{prol}}\cdot Prol\cdot\left( 1-\left( E_{\max}\cdot\frac{C_{\mathrm{RNP}}}{EC_{50}+C_{\mathrm{RNP}}} \right) \right)\cdot\left( \frac{\mathrm{Cir}c_{0}}{\mathrm{Circ}} \right)^{\gamma}-k_{\mathrm{tr}}\cdot Prol ; Prol_{0}=100$$

$$\frac{dT_{1}}{\mathrm{dt}}=k_{\mathrm{tr}}\cdot Prol-k_{\mathrm{tr}}\cdot T_{1}; T_{1_{0}}=100$$

$$\frac{\mathrm{dCirc}}{\mathrm{dt}}=k_{\mathrm{tr}}\cdot T_{1}-k_{\mathrm{circ}}\cdot Circ ; Circ_{0}=100$$

$$\frac{d_{\mathrm{LDL}}}{\mathrm{dt}}=k_{\mathrm{syn}}\cdot\left( \frac{\mathrm{Circ}}{\mathrm{Cir}c_{0}} \right)^{\gamma}-k_{\deg}\cdot LDL;LDL_{0}=100$$

**References**

1. Miyazawa K, Liu Y, Bazzazi H. Development of a minimal PBPK-QSP modeling platform for LNP-mRNA based therapeutics to study tissue disposition and protein expression dynamics. Frontiers in Nanotechnology. 2024;6.

2. Shah DK, Betts AM. Towards a platform PBPK model to characterize the plasma and tissue disposition of monoclonal antibodies in preclinical species and human. J Pharmacokinet Pharmacodyn. 2012;39(1):67-86.

3. Ayyar VS, Song D, Zheng S, Carpenter T, Heald DL. Minimal physiologically based pharmacokinetic-pharmacodynamic (mPBPK-PD) model of GalNAc-conjugated siRNA disposition and gene silencing in preclinical species and humans. Journal of Pharmacology and Experimental Therapeutics. 2021:JPET-AR-2021-000805.

4. Davies B, Morris T. Physiological parameters in laboratory animals and humans. Pharm Res. 1993;10(7):1093-5.

5. Sasaki Y, Iwama R, Sato T, Heishima K, Shimamura S, Ichijo T, et al. Estimation of glomerular filtration rate in conscious mice using a simplified equation. Physiol Rep. 2014;2(8).

6. Sternberg SH, Redding S, Jinek M, Greene EC, Doudna JA. DNA interrogation by the CRISPR RNA-guided endonuclease Cas9. Nature. 2014;507(7490):62-7.

7. Harwood HJ, Jr., Pellarin LD. Kinetics of low-density lipoprotein receptor activity in Hep-G2 cells: derivation and validation of a Briggs-Haldane-based kinetic model for evaluating receptor-mediated endocytotic processes in which receptors recycle. Biochem J. 1997;323 ( Pt 3)(Pt 3):649-59.
